# Epicardial fat remodeling in end-stage heart failure with reduced ejection fraction

**DOI:** 10.1186/s12933-026-03106-2

**Published:** 2026-02-15

**Authors:** Maciej Mączewski, Marta Załęska-Kocięcka, Maksymilian Nowakowski, Maciej Mazuruk, Łukasz Nogajski, Hanna Czerwińska, Igor Gronek, Mateusz Smoliński, Aleksandra Świstak, Mikołaj Kurpias, Oliwia Łuniewska, Marta Kacperska, Filip Pilzak, Zuzanna Wojdyńska, Ilona Michałowska, Michał Mączewski, Aleksandra Paterek, Przemysław Leszek

**Affiliations:** 1 Department of Clinical Physiology, Centre of Postgraduate Medical Education, Warsaw, Poland; 2 Student’s Cardiovascular Scientific Club “Kardioplegia”, Medical University of Warsaw, Warsaw, Poland; 3 Heart Failure and Transplantology Department, Mechanical Circulatory Support and Transplant Department, National Institute of Cardiology, Warsaw, Poland; 4 Department of Radiology, National Institute of Cardiology, Warsaw, Poland

**Keywords:** Heart failure with reduced ejection fraction, Epicardial fat, Computed tomography imaging, Adipose tissue, adipocytes

## Abstract

**Background:**

Epicardial adipose tissue (EAT) transformation in heart failure with reduced ejection fraction (HFrEF) is poorly understood, which limits its potential as a therapeutic target and prognostic factor. The aim of our study was to characterize EAT in patients with HFrEF at the histological, computed tomography (CT) imaging and radiomic level to better understand its transformation in HFrEF.

**Methods:**

We enrolled 70 patients with HFrEF who were scheduled for implantation of a left ventricular assist device (LVAD) or orthotopic heart transplantation (OHT). Fifty non–heart failure (HF) subjects served as controls. All participants underwent contrast- or non-contrast-enhanced chest CT imaging for EAT analysis. Left ventricular myocardial cones with overlying EAT were obtained from LVAD patients during surgery, from explanted OHT hearts, and from 20 unused healthy donor hearts for histological analysis.

**Results:**

While total EAT volume did not differ between non-HF and HFrEF subjects, its density assessed in CT images, was higher in HFrEF. Moreover, periventricular EAT exhibited density gradient, with the densest voxels immediately adjacent to the myocardium (over up to 1 mm). This density gradient was extended to almost 3 mm in LV EAT in HFrEF patients. Histological analysis showed that adipocytes also exhibited a characteristic cell size gradient, with smaller cells adjacent to the myocardium, more pronounced than in non-HF subjects; moreover, median LV EAT adipocyte size was smaller in HFrEF vs. non-HF patients. However, EAT fibrosis and blood vessel density did not differ between non-HF and HFrEF subjects. Both histological analysis and radiomic analysis of CT images revealed that EAT was more heterogeneous in HFrEF than in non-HF subjects. These changes were most pronounced in LV EAT, but other EAT depots (RV and periatrial) were also affected.

**Conclusions:**

LV EAT in HFrEF contains smaller adipocytes and has higher density in CT images, exhibits pronounced cell size/density gradient and is more heterogeneous than in non-HF subjects. Thus LV EAT undergoes complex remodeling in HFrEF. Further studies are needed to elucidate the mechanisms driving this remodeling, determine whether it can be therapeutically targeted, and assess which parameters may have prognostic value in patients with HFrEF.

**Supplementary Information:**

The online version contains supplementary material available at 10.1186/s12933-026-03106-2.

## Introduction

Epicardial adipose tissue (EAT) is a distinct fat depot interposed between the myocardium and epicardium, where its anatomic proximity enables paracrine, vasocrine, and direct cell–cell interactions that potentially modulate myocardial structure and function [1]. It can be a potential prognostic factor and target for therapies in multiple conditions, though currently both these areas are limited by our poor understanding of EAT (patho)physiology [2, 3].

EAT can be divided into specific compartments: pericoronary EAT (PC EAT), periatrial EAT (PA EAT) and periventricular EAT (PV EAT), respectively right (RV EAT) and left (LV EAT) ventricular. PC EAT undergoes pathological, pro-inflammatory transformation in coronary artery disease, especially around segments of epicardial coronary arteries with unstable, ruptured plaques or prone to rupturing [4], being a good predictor of coronary events [5, 6]. PA EAT also undergoes pathological, pro-inflammatory and pro-fibrotic transformation [7], potentially affecting the risk of atrial fibrillation (AF) [8] and is a good predictor of AF risk. Much less is known about PV EAT. In particular its biology is not well understood, mainly due to problematic access to the material.

PV EAT is especially important in the context of heart failure. In healthy individuals EAT volume correlates with impairment of diastolic [9] and systolic [10] LV function and is a predictor of coronary events [11] and incident heart failure [10]. Similarly in heart failure with preserved ejection fraction (HFpEF), EAT volume correlates with worse ventricular function [12] and adverse prognosis [13]. In contrast, the prognostic role of EAT in heart failure with reduced ejection fraction (HFrEF) remains controversial. One study reported that that greater EAT thickness assessed by echocardiography was associated with a lower risk of a composite outcome of heart failure hospitalization and cardiovascular death [14]. Conversely, another study found that higher EAT thickness was associated with a higher risk of a composite outcome of heart failure related death, heart failure hospitalizations and ventricular arrhythmias [15]. Moreover, high baseline EAT volume has been linked to a lower likelihood of improvement of LV function [16] and reverse remodeling [17]. These conflicting findings underscore the need for a deeper understanding of EAT biology in HFrEF, extending beyond its volume to include its composition and functional characteristics.

Our pilot study has previously shown that LV EAT undergoes specific transformation in HFrEF, characterized by increased density on CT imaging and reduced adipocyte size [18]. However, it remains unclear whether this transformation is restricted to the LV or generalized across total EAT, whether other components of EAT (e.g., collagen fibers, vasculature) also undergo remodeling, and how these changes relate to systemic factors, particularly metabolic, pro-inflammatory, and neurohumoral influences.

Thus the aim of this study was to characterize EAT in a large cohort of patients with end-stage HFrEF at histology, imaging and radiomic level to better understand its structure, function and possible transformation in HFrEF.

## Methods

The study complies with the *Declaration of Helsinki.* The local ethics committee has approved the research protocol (No. IK.NPIA.0021.28.2026/23) and informed consent has been obtained from each study subject. All data generated or analyzed during this study are included in this published article and its supplementary information files.

### Study population and material

The study arm 1 (HFrEF) consisted of 48 patients with end-stage HFrEF undergoing left ventricular assist device (LVAD) implantation (2 females/46 males) and 22 HFrEF patients undergoing orthotopic heart transplantation (OHT) (6 females/16 males).

The study arm 2 (non-HF) consisted of 50 subjects (8 females/42 males) undergoing computed tomography (CT) coronary angiography as part of the diagnostics for chest pain who presented normal left ventricular (LV) and right ventricular (RV) structure and function, had no significant stenoses of epicardial coronary arteries or any chronic diseases except adequately controlled hypertension, diabetes, atrial fibrillation or hypercholesterolemia (according to the attending physician’s judgment).

The study arm 3 (non-HF) consisted of 20 healthy hearts (4 female, 16 male) disqualified from transplantation due to non-cardiac causes.

A total of 70 patients from the study arm 1 underwent CT imaging of the chest within 7 days of respective cardiac surgical procedure. A LV myocardial cylinder with the overlying EAT was obtained from LVAD subjects during the surgery. A corresponding part of LV apical region was obtained from the explanted hearts (OHT subjects) and healthy hearts for further analyses (Arm 3).

Laboratory data were obtained from routinely performed blood tests.

### Computed tomography imaging and analysis of epicardial adipose tissue

CT scans were performed using a dual-source scanner (NAEOTOM Alpha, SOMATOM Force, Siemens Healthineers) with the use of iodine contrast agent. Detailed description can be found in Supplementary material.

**Fig. 1:**
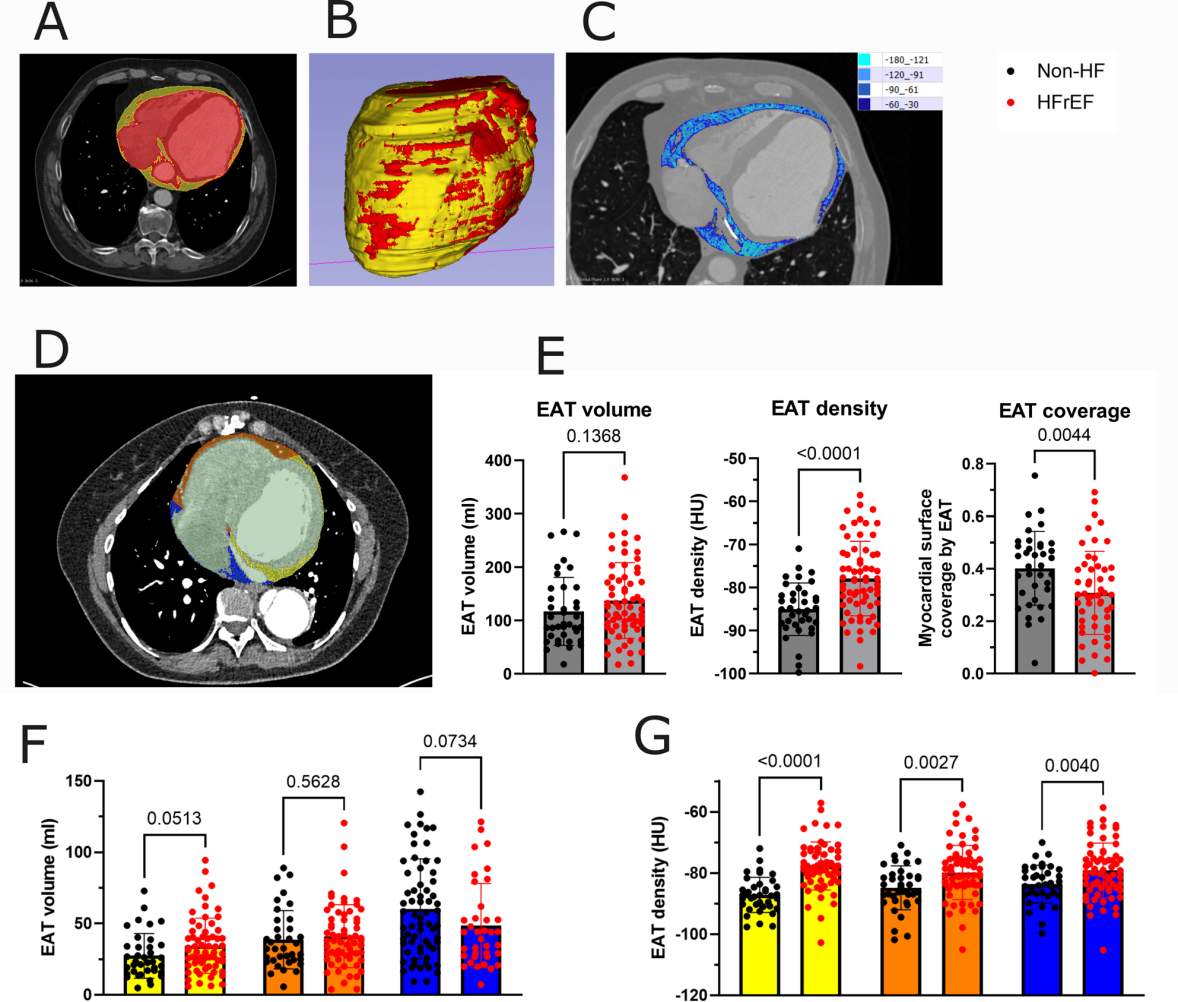
Characterization of epicardial adipose tissue in subjects with and without heart failure with reduced ejection fraction. **A** Epicardial adipose tissue (EAT) shown in yellow in a single slice of computed tomography (CT) image of the chest, as tissue with voxel density between − 30 and − 180 Hounsfield units (HU), between the border of the myocardium (shown in red) and manually traced parietal layer of the pericardium. **B** Three-dimensional reconstruction of EAT (in yellow) covering the myocardium (in red). **C** Color coded voxels of EAT (shades of blue, from dark blue corresponding to the highest density to light blue corresponding to the lowest density) in a single CT slice showing the densest EAT adhering to the myocardium. **D** Color coded EAT types: left ventricular (LV, yellow), right ventricular (RV, orange) and periatrial (PA, blue) (Student’s t test). **E** Total EAT median volume, density and myocardial surface coverage by EAT in non-heart failure (non-HF) subjects and heart failure with reduced ejection fraction (HFrEF) patients (Student’s t test). **F**-**G** LV, RV and PA EAT median volume (**F**) and density (**G**) in non-HF subjects and HFrEF patients. Dots represent medians for individual subjects, bars represent mean + standard deviation. Two-way ANOVA with post-hoc test

Pixel size ranged from 0.27⋅0.27 mm to 0.96⋅0.96 mm. Slice thickness was 0.4–0.6 mm. For calculating EAT density gradients only CT images with pixel size < 0.52⋅0.52 mm were used. Post-processing was done using 3D Slicer (Boston, USA, v. 5.2.2). EAT was defined as the fat tissue (density − 180 to − 30 Hounsfield units (HU)) [19] between the outer wall of the myocardium and the visceral layer of the pericardium. EAT density was measured as fat attenuation index in HU, with higher HU values indicating greater EAT density. Figure 1A and D demonstrate EAT delineation in a single ventricular slice, three-dimensional EAT reconstruction, various EAT density categories, and EAT subdivision into LV, RV and PA EAT, respectively. The myocardial surface coverage by EAT was expressed as the total surface area of EAT divided by the total epicardial surface. Pericardial adipose tissue (PAT) was obtained as total thoracic visceral fat with subtracted EAT.

### Radiomic analysis

Calculation of radiomic features was performed on CT scans of the chest using 3D Slicer for a single EAT slice at the level of the aortic root. For each segmentation, a total of 103 radiomic features were calculated, ranging from first order (distribution of individual voxels), through second order (texture features) to third order statistics, using the SlicerRadiomics extension which incorporates the Pyradiomics library into 3D Slicer (see Supplementary Table 1 for individual features).

### Histology

The LV myocardium with the overlying EAT was fixed in neutral buffered formalin, paraffin embedded and cut into 4 μm thick slides. Detailed description can be found in Supplementary material.

To obtain adipocyte area, slides were stained with hematoxylin and eosin and cells were manually traced using ImageJ software. For the assessment of LV EAT fibrosis, Picrosirius red staining was used. For the assessment of LV EAT blood vessel density, lectin staining (UEA-I #L8262 Sigma) was used.

### Statistical analysis

Data are shown as means±standard deviation (SD) or median±interquartile ranges, depending on distribution. The normality of data distribution was tested visually by histograms and QQ plots and using Shapiro-Wilk test. For statistical analyses of two groups exhibiting normal distribution, unpaired two-tailed *t*-test was used. For non-normally distributed data differences were analyzed by Mann-Whitney U test or Kruskal-Wallis test. For statistical analyses of three or more groups one-way or two-way analysis of variance (ANOVA) was used followed by post-hoc Tukey’s or Dunnett’s multiple comparisons tests. Correlation between variables was determined by Pearson’s correlation test. Owing to their non-normal distribution, NT-proBNP and CRP values were ln-transformed for the purposes of correlation analysis. Strength of correlations was based on correlation coefficients as: ≥0.60 strong, 0.40–0.60 moderate and < 0.40 weak. Multivariate linear regression analysis was used to examine the association between LV EAT volume/density and a set of continuous (BMI, ln-BNP, ln-CRP) and categorical (e.g., CAD, AF, diabetes mellitus) explanatory variables. A P value of less than 0.05 was considered statistically significant. Bonferroni correction for multiplicity was used for radiomic parameters. Information on the group size, statistical analysis used as well as P-values is provided in the figure captions. Sigma Plot 15.0 (Inpixon, CA, USA) was used for statistical analyses.

## Results

### Characterization of epicardial adipose tissue by computed tomography in subjects with and without HFrEF

Table 1 presents demographic and clinical characteristics of the study subjects with and without HFrEF. Mean age, sex distribution and body mass index (BMI) as well as incidence of hypertension and diabetes mellitus did not differ between the study groups. The dominant HFrEF etiology (53%) was dilated cardiomyopathy.

**Table 1:** Demographic data of the study cohorts

Cohort	HFrEF (*n* = 70)	Non-HF control subjects (*n* = 70)			*p* for HFrEF vs. non-HF comparison
Subdivision	LVAD (*n* = 48) and OHT (*n* = 22)	All (*n* = 70)	Control subjects for CT imaging (*n* = 50)	Healthy heart donors (*n* = 20)	
Age, years	51.5±11.3	57.0±15.0	59.0±16.1	38.9±15.1	0.11
Sex, M/F	62/8	58/12	42/8	16/4	0.35
Ethnicity (White)	70 (100%)	70 (100%)	50 (100%)	20 (100%)	1.00
Body mass index kg/m^2^	28.1±5.3	27.0±4.7	27.1±4.5	26.8±5.4	0.21
HFrEF etiology					
DCM	37 (52.9%)				
ICM	27 (38.6%)				
Other	6* (8.6%)				
Diabetes	17 (24.3%)	12 (17.1%)	11 (22.0%)	1 (5.0%)	0.30
Hypertension	20 (28.6%)	17 (24.3%)	14 (28.0%)	3 (15.0%)	0.56
Atrial fibrillation	29 (40.2%)	5 (7.1%)	3 (8.0%)	2 (10.0%)	< 0.0001
Coronary artery disease	30 (42.9%)	0	0	0	< 0.0001
Implantable device (pacemaker, ICD, CRT)	49 (70.0%)	1 (1.8%)	1 (2%)	0	< 0.0001
LVEF, %	17.6±6.6	58.5±6.2	58.0±6.7	59.1±4.5	< 0.0001
LVEDD, mm	73.8±9.3	50.1±4.5	50.2±5.5	48.1±3.4	< 0.0001
LVM, g	320±66	138±51	141±48	135±33	< 0.0001
Treatment					
ACE-I	22 (31.4%)	16 (22.9%)	14 (28.0%)	2 (10.0%)	0.25
ARB	6 (8.6%)	6 (8.6%)	6 (12.0%)	0	1
ARNI	30 (42.9%)	0	0	0	< 0.0001
Beta blocker	66 (94.3%)	15 (21.4%)	14 (28.0%)	2 (10.0%)	< 0.0001
SGLT2-i	61 (87.1%)	6 (8.6%)	6 (12.0%)	0	< 0.0001
Statin	50 (71.4%)	8 (11.4%)	8 (16.0%)	0	< 0.0001
Metformin	15 (21.4%)	7 (10.0%)	7 (14.0%)	0	0.063
Insulin	5 (7.1%)	0	0	0	0.062
Pressor amines	23 (32.9%)	0	0	0	< 0.0001

*complex aortic valve defect (3), non-compaction (2), hypertrophic cardiomyopathy (1)

Results are presented as means ± standard deviations or as counts (percentages). P values were calculated using Student’s t test or chi-square/Fisher’s exact test, as appropriate. ACE-I, angiotensin converting enzyme inhibitor; ARB - angiotensin receptor blocker; ARNI, angiotensin receptor neprilysin inhibitor; CRT, cardiac resynchronization therapy; CT, computed tomography; DCM, dilated cardiomyopathy; HFrEF, heart failure with reduced ejection fraction; ICD, implantable cardioverter-defibrillator; ICM, ischemic cardiomyopathy; LVAD, left ventricular assist device; LVEF, left ventricular ejection fraction; LVEDD, left ventricular end diastolic diameter; LVM, left ventricular mass; OHT, orthotopic heart transplantation; SGLT2-i, sodium glucose cotransporter 2 inhibitor

Total EAT volume assessed with CT imaging, did not differ between subjects with and without HFrEF (Fig. 1E). No differences were also found for LV, RV or PA EAT volumes (Fig. 1F). However, myocardial surface coverage by EAT was reduced in HFrEF subjects, reflecting LV dilation and increased myocardial surface (Fig. 1E). Indeed, LV EAT volume corrected for LV weight was almost halved in HFrEF versus non-HF patients (0.42±0.24 ml/g vs. 0.79±0.47 ml/g, respectively, *p* < 0.01).

Total EAT density, measured as fat attenuation index, was higher in HFrEF patients (Fig. 1E). EAT density was higher in HFrEF subjects for the LV EAT, while only mildly elevated also for RV EAT and PA EAT (Fig. 1G).

**Fig. 2:**
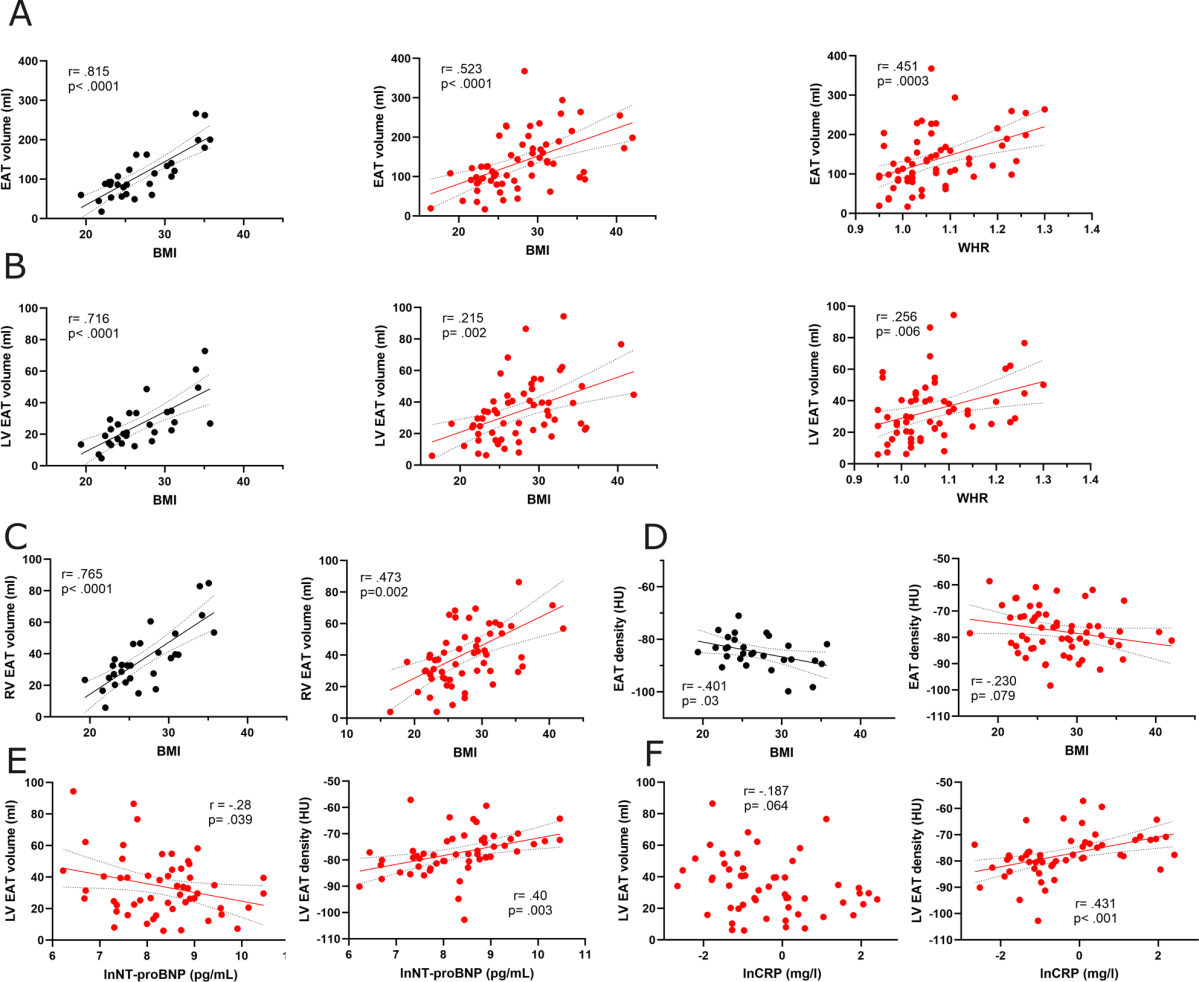
Correlation between epicardial adipose tissue characteristics and systemic metabolic, inflammatory, neurohormonal and other parameters in subjects with and without heart failure with reduced ejection fraction. Correlation between total (**A**), left ventricular (LV) (**B**) and right ventricular (RV) (**C**) epicardial adipose tissue (EAT) volume or density (**D**) and body mass index (BMI) or waist to hip ratio (WHR) in non-heart failure (non-HF, black dots and lines) subjects and heart failure with reduced ejection fraction (HFrEF, red dots and lines) patients. Correlation between LV EAT volume and density and ln-transformed serum NTpro-BNP (**E**) and C-reactive protein (CRP) (**F**) concentration. All panels display individual data points and the results of Pearson’s correlation analysis, with r indicating correlation strength and p indicating statistical significance for each association; dotted lines represent the confidence intervals

Total, LV EAT and RV EAT EAT volumes were closely correlated with body mass index (BMI) in non-HF subjects, whereas the strength of these correlations was substantially weaker in patients with heart failure (Fig. 2A-C). To test the hypothesis that BMI may poorly reflect overall adiposity in the HFrEF cohort, we examined waist-to-hip ratio (WHR) as an alternative index of obesity; however, the correlations between WHR and total or LV EAT volume were nearly identical to those observed with BMI (Fig. 2A-B). EAT density showed a moderate negative correlation with BMI in non-HF subjects, whereas no such association was observed in HFrEF patients (Fig. 2D).

**Fig. 3:**
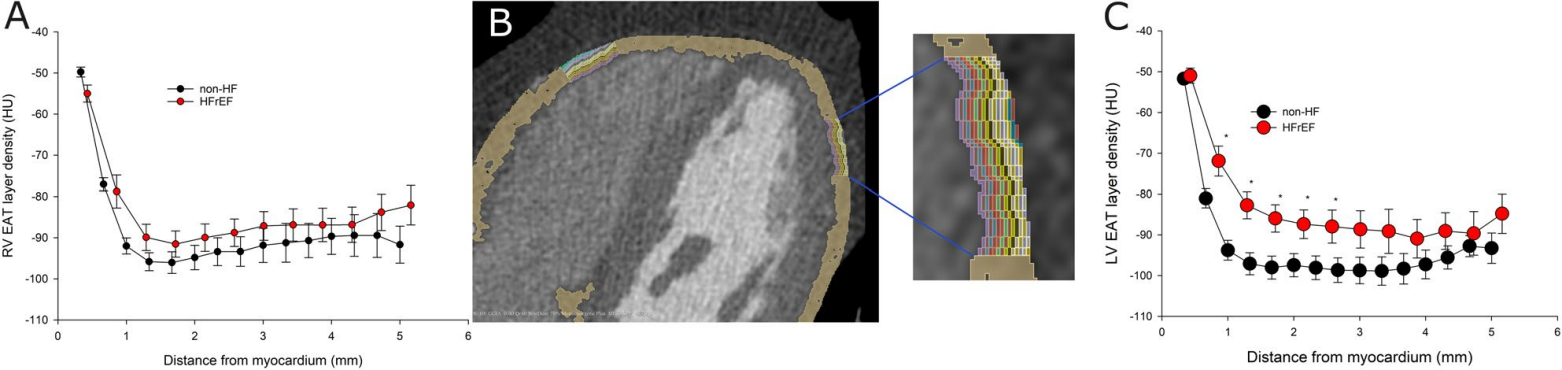
Characterization of epicardial adipose tissue density gradient in computed tomography images in subjects with and without heart failure with reduced ejection fraction. **A**-**C** Left ventricular (LV) and right ventricular (RV) epicardial adipose tissue (EAT) density gradients as a function of distance from the myocardium in non-heart failure (non-HF) subjects and heart failure with reduced ejection fraction (HFrEF) patients. Each data point indicates a mean voxel density in a specific layer. n = 50 for non-HF subjects and n = 61 for HFrEF patients. Two-way ANOVA with post hoc Tukey’s test. **B** Epicardial adipose tissue (EAT) marked in dark yellow with color coded individual voxel layers

However, both total, LV and RV EAT volume and density exhibited weak, but significant correlations with serum ln-transformed NT-proBNP, negative and positive, respectively (Fig. 2E, S1). Moreover, weak though significant correlation was found between a systemic inflammatory marker, CRP, and EAT density, but not volume (Fig. 3F, S1). Pericardial adipose tissue (Peri AT, considered a visceral fat depot) volume and density strongly correlated with total EAT volume and density, respectively (Fig.S1) both in non-HF and HFrEF patients.

In a multivariable linear regression analysis of LV EAT that included BMI, ln-transformed NT-proBNP and CRP, as well as comorbidities such as coronary artery disease, atrial fibrillation, and diabetes mellitus, BMI was the only significant explanatory variable associated with LV EAT volume, whereas ln(CRP) was the only significant explanatory variable associated with LV EAT density (Table S1).

### Epicardial adipose tissue density gradients

Detailed analysis of CT scans from non-HFrEF subjects revealed that EAT exhibited clear density gradient over both LV and RV, i.e. two first layers of voxels corresponding to EAT located closest to the myocardium (i.e. within 0.6–0.7 mm), exhibited higher density, while more distant EAT had lower and largely homogeneous density (Fig. 3A-C).

However, in HFrEF patients the LV EAT density gradient extended over up to 2.5 mm from the myocardium (Fig. 3C), i.e. within this space EAT over the failing LV was clearly denser than EAT over the healthy LV. No such differences were found between HFrEF and non-HF regarding RV EAT density gradients (Fig. 3A). Moreover, EAT density gradients did not differ between individual chambers in non-HF or HFrEF patients.

### Adipocyte size, fibrosis and blood vessel density in left ventricular epicardial adipose tissue

**Fig. 4:**
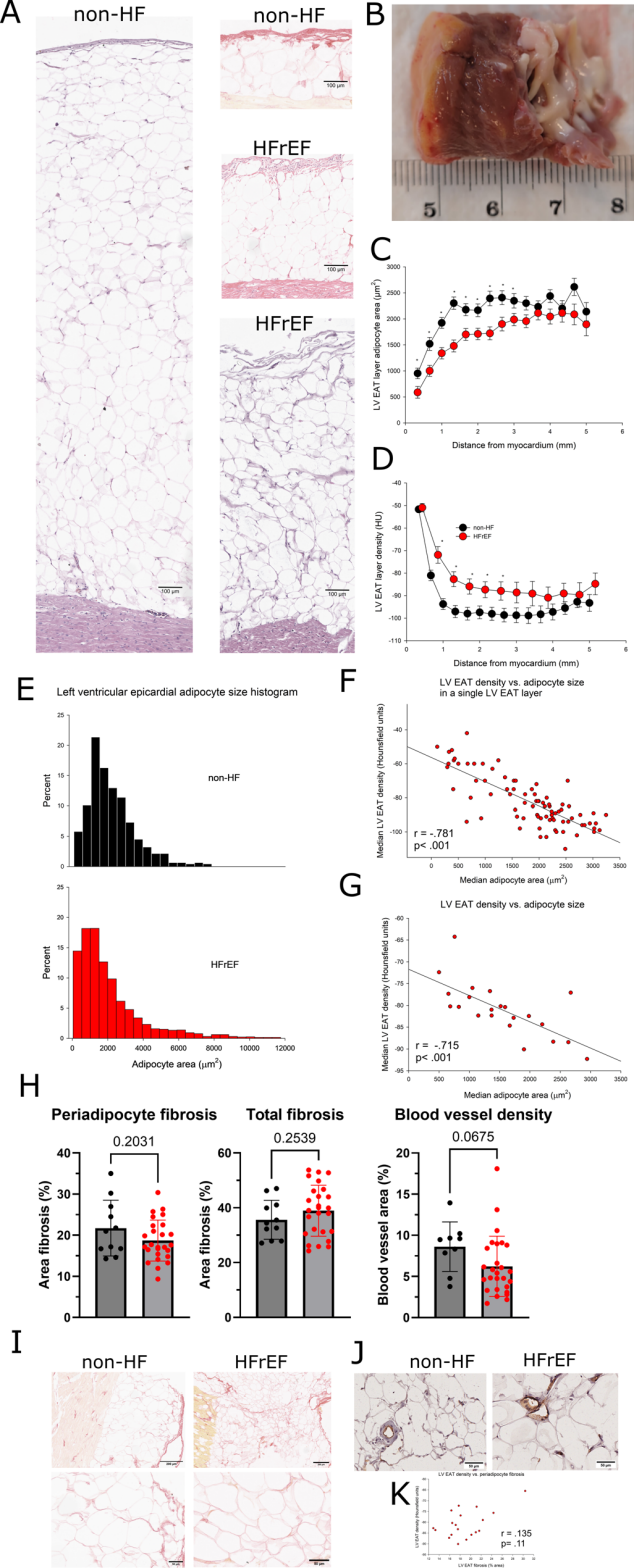
Left ventricular epicardial fat histology in subjects with and without heart failure with reduced ejection fraction. **A** A representative hematoxylin and eosin stained histology image of left ventricular (LV) epicardial adipose tissue (EAT) from a non-heart failure subject with large (left) and small (middle upper) volume of EAT and from a heart failure with reduced ejection fraction (HFrEF) patient with small (middle centre) and large (middle lower) volume of EAT. **B** Cylindrical tissue sample from the left ventricle, collected during LVAD implantation, along with EAT layer on the left. **C** Adipocyte area gradients from histological analysis. n = 20 for non-heart failure (non-HF) and n = 48 for HFrEF patients and corresponding **D** LV EAT density gradients obtained from CT images (redrawn from Fig. 3C) with Two-way ANOVA with post hoc Tukey’s test. **E** LV EAT adipocyte size histogram from non-HF (n=20) and HFrEF (n=48) patients. **F** Correlation between median adipocyte area in a specific LV EAT layer and median LV EAT voxel density in the same layer in a CT image from the same patient. **G** Correlation between median adipocyte area in LV EAT from a single patient and median LV EAT voxel density in a CT image from the same patient. Pearson’s correlation test. **H** Median periadipocyte, total fibrosis in LV and median blood vessel area in LV EAT. Dots represent medians for individual subjects, bars represent mean + standard deviation. Student’s t test. **I** Representative images of Picrosirius red stained LV EAT fibrosis in a non-HF subject and HFrEF patient. **J** Representative images of lectin stained LV EAT blood vessels in a non-HF subject and HFrEF patient. **K** Correlation between median fibrosis area in LV EAT and median LV EAT voxel density in CT image from the same patient. Pearson’s correlation test

Histological analysis (Fig. 4A-B) revealed that all samples of LV EAT from both non-HF and HFrEF subjects were composed of exclusively white adipose tissue, with no histological evidence of brown or beige adipocytes (white adipose tissue can be reliably identified on routine H&E staining by the presence of large unilocular adipocytes containing a single large lipid droplet, scant cytoplasm, and a flattened, peripherally displaced nucleus. In contrast to brown adipose tissue, white adipocytes lack multilocular lipid droplets and have less abundant cytoplasm). In LV EAT from non-HF subjects the smallest adipocytes were found in the vicinity of the myocardium, while the number of small adipocytes decreased and median adipocyte size increased with distance from the myocardium, reaching plateau approximately 0.7 mm from the myocardium (Fig. 4C). When we divided the EAT into approximately 300 μm layers corresponding to an average pixel size in CT images, we found an adipocyte size gradient corresponding to CT density gradient. In HFrEF median adipocyte size increased more gradually with distance from the myocardium, corresponding to the density gradient in CT images (Fig. 4D). The difference in median adipocyte size between subjects with and without HFrEF persisted up to 3 mm from the myocardium. Median LVEAT adipocyte size was smaller in subjects with than without HFrEF (1714 vs. 2214µm^2^, respectively, *p* < 0.01). Distribution of adipocyte size in LV EAT revealed marked leftward shift in HFrEF subjects, but surprisingly also increased rate of giant adipocytes (> 8000µm^2^) (Fig. 4E) was found. Thus LV EAT in HFrEF was more heterogeneous with regard to adipocyte size, containing multiple small adipocytes, but also a sizeable fraction of giant adipocytes.

There was a strong correlation between a median voxel density in a given layer in the CT image and median adipocyte size in a corresponding histological layer of LV EAT from the same patient (Fig. 4F). A similar correlation was observed between median LV EAT density in the CT image and median LV EAT adipocyte size (Fig. 4G), supporting the concept that EAT density reflects median adipocyte size and that reduced LV EAT density in the close vicinity of the myocardium is a reflection of reduced median adipocyte size in this region.

To check for other factors potentially affecting density of LVEAT, we analyzed fibrosis and blood vessels in LV EAT, two other major components of adipose tissue, in subjects with and without HFrEF, finding no differences in either total or periadipocyte fibrosis (Fig. 4H). The third significant component of adipose tissue, blood vessels (Fig. 4H), also did not differ between HFrEF and non-HFrEF subjects, supporting our observation that main difference between healthy and HFrEF LV EAT lies in adipocyte size. Representative images of LV EAT fibrosis and blood vessels are provided in Fig. 4I-J. There was no correlation between LV EAT density and the degree of LV EAT fibrosis (Fig. 4K), indicating that adipose tissue density is predominantly a reflection of its adipocyte size at the histological level.

### Radiomic analysis of epicardial fat

**Fig. 5:**
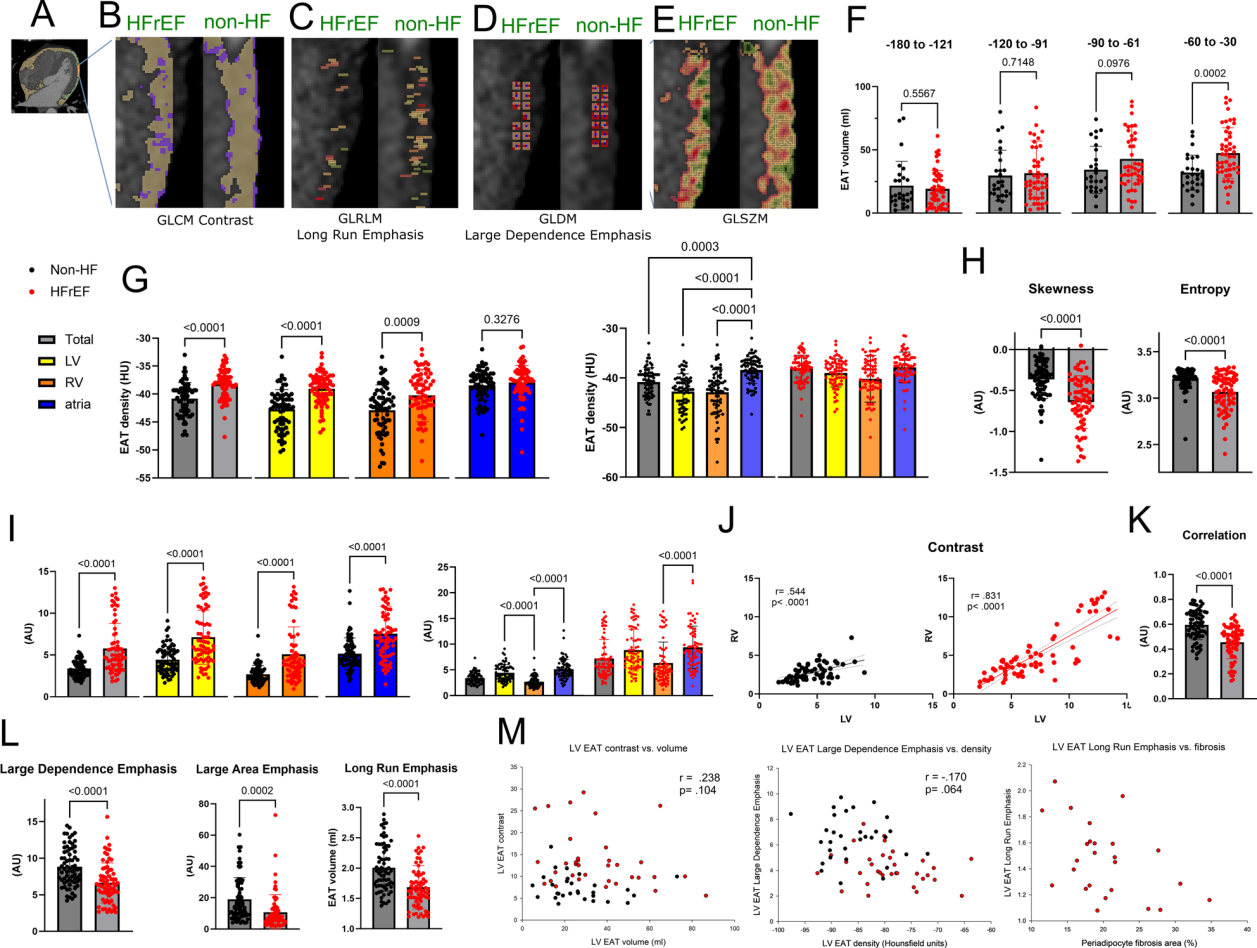
Radiomic features of left ventricular epicardial fat in subjects with and without heart failure with reduced ejection fraction. **A** A sample computed tomography of the chest slice through the right and left ventricle (its lumen filled with white contrast, short axis view) and segmented epicardial fat: dark yellow around the right and green around the left ventricle with the investigated fragment marked between the two blue lines. **B** A fragment of segmented left ventricular periventricular epicardial adipose tissue (LV EAT) from a HFrEF subject (on the left) and a non-HF subject (on the right) with instances of 2 adjacent voxels that differ with density > 10 Hounsfield units, a measure of contrast, shown in violet. While in a non-HF subject these violet areas group near the myocardium and the epicardium, in a HFrEF patient there is a significant presence of contrast inside LV EAT tissue. **C** Gray Level Run Length Matrix (GLRLM) assesses the length and count of uninterrupted voxel runs with the same intensity along a given direction. The feature Long Run Emphasis (LRE) increases when longer runs are more common, indicating greater tissue homogeneity. Runs of ≥ 3 voxels along the x-axis are marked. Clearly LRE is higher in a non-HF individual. **D** Gray Level Dependence Matrix (GLDM) measures the number of neighboring voxels with similar intensities. The feature Large Dependence Emphasis (LDE) increases when there are many voxels surrounded by multiple neighbors with similar HU values, indicating greater tissue homogeneity. Here neighbors within ± 10 Hounsfield units of the central voxel are marked in dark red. Again, LDE is higher in the non-HF LV EAT. **E** Gray Level Size Zone Matrix (GLSZM) quantifies the number and size of homogeneous zones—groups of connected voxels with the same intensity. The feature Large Area Emphasis (LAE) increases with more large, uniform regions. These zones are represented as regions of the same color in the visualization. LAE is higher in non-HF subjects in the sample image, again a marker of greater tissue homogeneity. **F** EAT volume in specific density ranges, from the lowest density on the left to the highest on the right. **G**-**H** First order radiomic parameter: 90 percentile and its distribution over specific cardiac chambers as well as skewness and entropy. **I**-**K** Second order radiomic parameters: contrast and its distribution over specific cardiac chambers, correlation between LV and RV contrast in non-HF (left) and HFrEF (right) patients as well as correlation. **L** Higher order radiomic parameters, Mann Whitney’s test with Bonferroni correction for multiplicity. **M** Correlation between specific radiomic parameters and LV EAT volume, density and fibrosis. Pearson’s correlation test

To further characterize LV EAT from failing hearts, we performed radiomic analysis of CT images (Fig. 5A-E). Detailed description of radiomic parameters can be found in Fig. 5 and in Supplementary materials (Table S2).

As expected from higher median EAT density, volume of the densest EAT was higher in HFrEF than in non-HF subjects, while the other voxel categories did not differ (Fig. 5F). First order radiomics confirmed shift of EAT voxels to more dense values, but this was found only in LV and RV, but not PA EAT (Fig. 5G). Indeed, while in non-HFrEF subjects least dense EAT was found around LV and RV, while the densest in the atria, in HFrEF this difference almost disappeared (Fig. 5G). Other first order radiomic parameters also differed between HFrEF and non-HF patients (Fig. 5H). However, while increased skewness, a measure of the asymmetry of the distribution of voxel intensities, was expected in HFrEF patients, reduced entropy, a measure of randomness of voxel intensity values within a region of interest, without considering spatial relationships, was an unexpected finding (Fig. 5H).

Second order radiomics, describing the spatial arrangement of patterns, revealed that non-HF LV EAT was more homogeneous, i.e. contrast was higher and correlation was lower in HFrEF than in non-HF for LV, RV and PA EAT (Fig. 5I-K). Again, intrinsic differences between specific EAT compartments in contrast tended to disappear in HFrEF (Fig. 5K). Moreover, correlation between LV and RV EAT contrast was much stronger in HFrEF, than in non-HF patients (Fig. 5J).

Higher order radiomics, characterizing more complex spatial patterns, confirmed that non-HF EAT was more homogeneous (Fig. 5L).

Second or higher order radiomic parameters did not correlate with either LV EAT volume, density or fibrosis (Fig. 5M), suggesting that they provide added information on adipose tissue structure, unavailable to conventional methods.

## Discussion

**Fig. 6:**
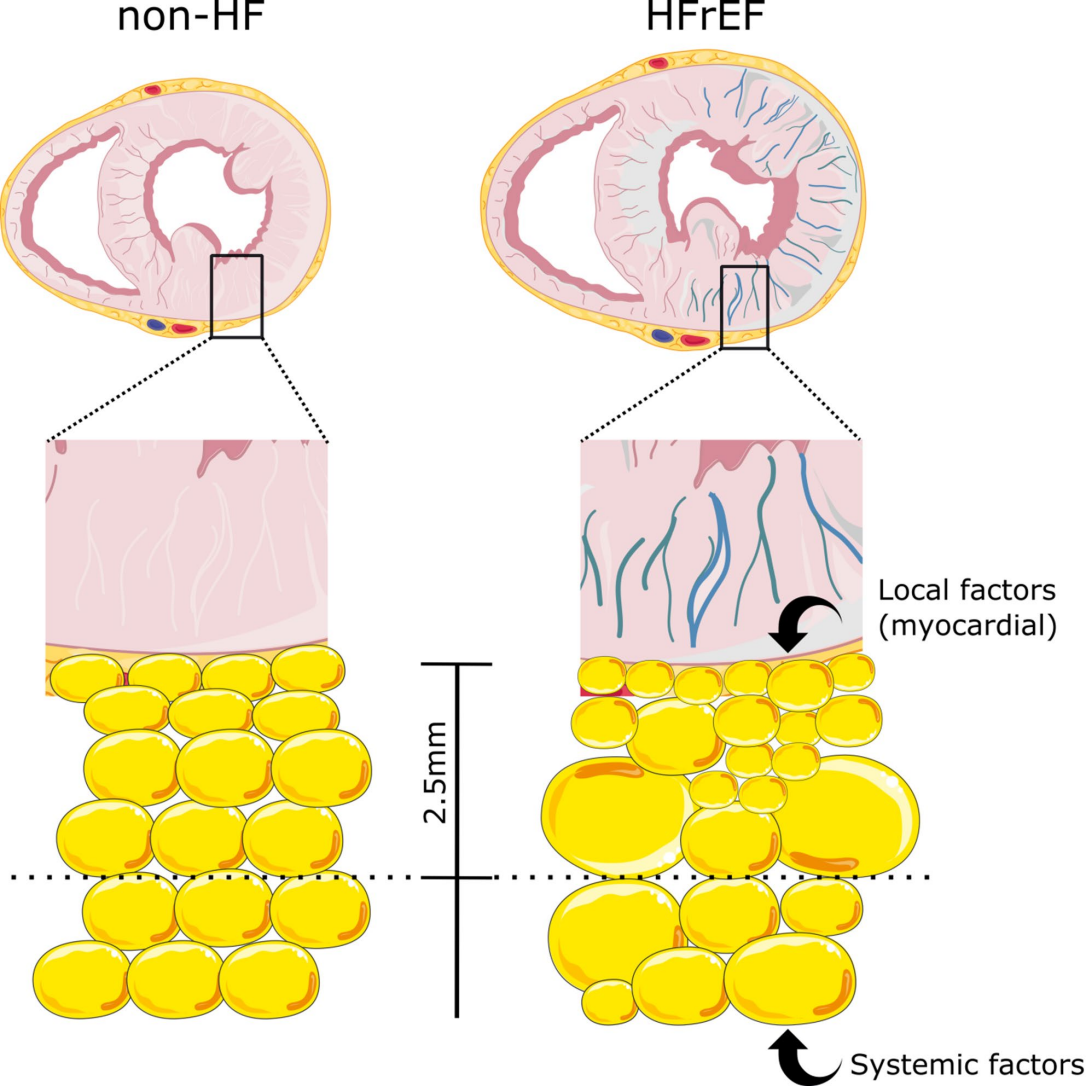
Left ventricular epicardial adipose tissue remodeling in heart failure with reduced ejection fraction. Complex remodeling of left ventricular (LV) epicardial tissue (EAT) in patients with heart failure with reduced ejection fraction (HFrEF), involving two distinct components: (1) a systemic component, which similarly affects all EAT depots and likely reflects neurohumoral activation, systemic inflammation, and cachectic signaling — hallmarks of HFrEF; and (2) a local component, specific to the LV depot, in which only the EAT layer adjacent to the failing LV is selectively altered

Here we show LV EAT remodeling in HFrEF that involves (1) increased density in CT images, which reflects smaller adipocyte size in histological analyses, (2) these effects are most pronounced in the innermost 2.5 mm EAT layer immediately adhering to the myocardium; moreover (3) LV EAT becomes more heterogeneous, both in micro scale, as reflected by higher dispersion of individual adipocyte size and in macro scale, as reflected by radiomic parameters. However these changes are not limited to LV EAT, but also occur in RV and PA EAT, though to a lesser degree. This suggests that in HFrEF there are two sources triggering EAT remodeling: an external, systemic one, that probably equally affects all EAT depots, and an internal one, originating from the failing LV, that preferentially affects LV EAT (Fig. 6). Thus our study indicates that EAT remodeling in HFrEF reaches far beyond changes of total EAT volume and density and its sources are complex. Since EAT has been proposed as a prognostic factor and possible target for therapies, understanding of its complex pathophysiology and transformation in HFrEF becomes an urgent need.

In healthy subjects metabolic factors, such as visceral obesity, BMI correlate with total EAT volume [20]. In HFpEF total EAT volume is increased, but the correlation between EAT and BMI is preserved, as in healthy subjects [21–23]. A recent metaanalysis [24] included 6 studies that assessed EAT volume using either CT or magnetic resonance imaging and found that it is unchanged in patients with HFrEF compared with healthy controls, whereas total EAT mass per gram of cardiac tissue is reduced [25–32] and correlation between BMI and total EAT is lost [27, 31]. In our cohort, we demonstrate that in HFrEF, the correlation between LV EAT volume or density and BMI is weakened or completely lost, suggesting that other factors contribute to LV EAT biology. We can speculate that systemic neurohumoral (NT-proBNP) and inflammatory mediators (indicated by CRP) may play a role here, based on their correlation with LV EAT volume and/or density. These systemic processes appear to affect all EAT depots — not only LV EAT, but also RV and PA EAT.

For the first time we show here that there may be a second component of EAT remodeling in HFrEF, unique for the EAT depot over the failing LV. In our analysis LV EAT in HFrEF exhibits extended density gradient in CT images, up to 2.5 mm from the myocardial border, mirroring adipocyte size gradient by histology, showing no correlation with either fibrosis or blood vessel density. Similar gradients were found for other EAT compartments: PC EAT and PA EAT. However, each EAT compartment seems to have its unique specificity: adipocyte size and density gradient in PC EAT persists over much larger distance (up to 15 mm vs. 2.5 mm in our study) [4]. In the PC EAT this gradient is a reflection of local inflammation originating in the coronary artery [4]. It is unknown if the same is true in the LV EAT, but if so, the strength of this interaction seems to be much weaker, as suggested by shorter distance of adipocyte size gradient. On the other hand, in PA EAT such adipocyte size gradient, as in our LV EAT, reaches up to 2–3 mm from the atrial myocardium, however PA EAT exhibits much more fibrotic remodeling associated with adipocyte size [33]. Here we confirm the finding observed for PC EAT [4] that higher density in CT images reflects smaller adipocytes in histological examination. Balance between lipid (low density) and aqueous phase (high density) is driven by adipocyte size due to accumulation of fat droplets in large adipocytes [34]. This observation is supported by the fact that we found no brown on beige adipocytes in EAT from non-HF or HFrEF subjects and found no correlation between either LV EAT fibrosis or blood vessels and its density.

Last but not least, we show that EAT transformation in HFrEF is also reflected by its greater heterogeneity as compared to non-HF subjects. Specifically, while in healthy LV EAT adipocytes are fairly uniform with the exception of their first 4–5 layers immediately adjacent to the LV myocardium, LV EAT in failing hearts not only exhibits extended adipocyte size gradient over up to 30–40 layers of adipocytes, but also its distant adipocytes tend to be less uniform: there are multiple tiny cells (surface area < 500 µm^2^) as well as giant ones (> 8000 µm^2^). Radiomic analysis demonstrates that CT image of EAT also exhibits greater heterogeneity, i.e. groups of 30–40 adipocytes corresponding to a single voxel in a CT image, also are less homogeneous in HFrEF vs. non-HF EAT. Since this radiomic heterogeneity does not correlate with LV EAT volume, density or fibrosis, it represents another layer of EAT heterogeneity that is currently not well understood.

Overall, our findings suggest that EAT may undergo multifaceted remodeling in patients with HFrEF, involving two potentially distinct components. First, a systemic component appears to affect EAT across all depots (LV, RV, and PA), presumably reflecting broader processes such as neurohumoral activation, systemic inflammation, and cachexia that characterize HFrEF. Second, a more localized component seems to be present in the LV depot, where the EAT layer adjacent to the failing LV shows selective alterations. Further studies will be needed to clarify the underlying mechanisms of these changes and to explore whether they may represent viable therapeutic targets. In addition, future investigations should assess which aspects of EAT remodeling carry prognostic significance in patients with HFrEF.

### Limitations

Our study predominantly included White men, reflecting the typical Polish end-stage HFrEF population; however, this limits the generalizability of our findings to other demographic groups. In addition, participants were recruited from among LVAD and OHT candidates and were therefore younger than the broader HFrEF population. The cross-sectional design represents another limitation. Furthermore, histological analyses were confined to LV EAT, whereas assessments of other EAT depots relied solely on CT imaging. Finally, although CT imaging and histological samples were obtained from the same patients with HFrEF, different non-HF comparator groups were used: CT images were derived from non-HF patients undergoing coronary CT angiography, while histological samples were obtained from healthy donor hearts not used for transplantation.

## Supplementary Information

Below is the link to the electronic supplementary material.

Supplementary Material 1

## Data Availability

No datasets were generated or analysed during the current study.
