# Supplementary material for: Epicardial fat remodeling in end-stage heart failure with reduced ejection fraction
Source: Cardiovasc Diabetol. 2026 Feb 15;25:93. doi: 10.1186/s12933-026-03106-2 (PMC13011763; doi:10.1186/s12933-026-03106-2)
Supplement: Supplementary file 1 — Supplementary Material 1 [file 12933_2026_3106_MOESM1_ESM.docx]

**Methods**

***Computed tomography imaging and analysis of epicardial adipose tissue***

CT scans were performed using dual-source scanners (**NAEOTOM Alpha, SOMATOM Force, Siemens Healthineers**) either with an iodine-based contrast agent (all 50 non-HF and 58 HFrEF subjects) or without it (12 HFrEF subjects). Because no differences in EAT volume or density were observed between contrast-enhanced and non–contrast-enhanced images, both image types were included in the analysis. Non-contrast images were routinely acquired by a prospectively ECG-triggered high-pitch spiral acquisition mode followed by a retro­spectively ECG-gated CT scans with contrast. The following parameters were used: gantry rotation time 250ms; tube voltage, 90-140 kV (NEATOM) and 70–110 kV (SOMATOM), slice collimation of 192×0.6 mm (SOMATOM), 144x0.4x2 (NEATOM), tube currents 550–600 mAs (SOMATOM). Pixel size ranged from 0.27×0.27 mm to 0.96×0.96 mm. Slice thickness was 0.4 or 0.6 mm. For calculating EAT density gradients only CT images with pixel size <0.52×0.52 mm were used. To achieve heart rate of 65–75 bpm metoprolol in fractionated doses of 2.5 mg was injected intravenously.

Post-processing was done using 3D Slicer (Boston, USA, v. 5.2.2). EAT was defined as the fat tissue (density −180 to −30 Hounsfield units (HU)) between the outer wall of the myocardium and the visceral layer of the pericardium, which was quantified using a region of interest (ROI) manually defined by tracing the pericardium from the upper edge of the right auricle to the LV apex. Then the software reconstructed EAT into a three-dimensional region and automatically measured EAT volume and median attenuation by including contiguous three-dimensional fat voxels in the ROI. EAT was subsequently divided into three compartments: right ventricular (RV EAT), left ventricular (LV EAT) and atrial (Atrial EAT), using semi-automatic outlining of these chambers. To create a density gradient, a part of RV and LV EAT was marked on multiple slices (1.5–2 cm in length). Then, the first layer (one voxel wide) was designated in contact with the myocardium and subsequent one-voxel layers were semi-automatically marked across the entire EAT width. Subsequently median density of each layer was calculated. To eliminate the potential influence of PCAT on EAT density, this segmentation was done at least 2 cm from main branches of coronary arteries. Figures 1A-1C demonstrate EAT delineation in a single ventricular slice, three-dimensional EAT reconstruction and various EAT density categories, respectively.

***Histology***

The LV myocardium with the overlying EAT was fixed in neutral buffered formalin, paraffin embedded and cut into 4 µm thick slides. Slides were stained and scanned at x40 magnification using a NanoZoomer S210 (Hamamatsu) digital slide scanner.

To obtain adipocyte area, slides were stained with hematoxylin and eosin and cells were manually traced using ImageJ software. EAT was divided into 300 µm zones corresponding to an average 300 µm voxel size in CT images. At least 50 adipocytes were assessed in a single layer and median adipocyte area was calculated for each layer.

For the assessment of EAT fibrosis, Picrosirius red staining was used. Periadipocyte fibrosis was calculated using ImageJ software as fibrosis immediately surrounding specific adipocytes. Total fibrosis included areas of epicardium and around blood vessels. Fibrosis was expressed as a percentage of the area stained in red to EAT area.

For the assessment of EAT blood vessel density, lectin staining (UEA-I #L8262 Sigma) followed by streptavidin-HRP was used. Visualization was performed using DAB as a chromogen. Sections were counterstained with hematoxylin. Blood vessels were identified as lectin-positive areas, and only those with a diameter of ≤500 µm were included in the analysis. The total area occupied by these blood vessels was quantified using ImageJ and expressed as a percentage of the total EAT area.

**Supplementary Table 1.** Multiple linear regression analysis of left ventricular epicardial fat in HFrEF patients

| **Explanatory variable** | **β** | **95% CI** | **p-value** |
| --- | --- | --- | --- |
| Volume |  |  |  |
| ln(CRP) | -2.653 | -6.777 to 1.470 | 0.2018 |
| ln(BNP) | -0.9691 | -6.477 to 4.539 | 0.7249 |
| ***BMI (kg/m²)*** | ***1.753*** | ***0.8373 to 2.668*** | ***0.0004*** |
| CAD | -2.169 | -11.49 to 7.150 | 0.6418 |
| AF | -4.789 | -14.08 to 4.506 | 0.3053 |
| DM | 9.190 | -0.1950 to 18.58 | 0.0548 |

Density

| ***ln(CRP)*** | ***2.454*** | ***0.5996 to 4.309*** | ***0.0106*** |
| --- | --- | --- | --- |
| ln(BNP) | 2.052 | -0.4253 to 4.529 | 0.1023 |
| BMI (kg/m²) | 0.062 | -0.3498 to 0.4737 | 0.7634 |
| CAD | 1.337 | -2.855 to 5.528 | 0.5243 |
| AF | 1.486 | -2.695 to 5.667 | 0.4781 |
| DM | -1.624 | -5.846 to 2.597 | 0.4428 |

AF, atrial fibrillation; BMI, body mass index; BNP, brain natriuretic peptide; CAD, coronary artery disease; CRP, C-reactive protein; DM, diabetes mellitus; ln, natural logarithm transformed;

**Supplementary Table 2.** Radiomic data characterizing epicardial adipose tissue (EAT) in HFrEF (n=70) and non-HF (n=50) subjects

| **Category** | **Feature** | **Non-HF mean** | **SD** | **HFrEF mean** | **SD** | **t test non-HF vs. HFrEF** |
| --- | --- | --- | --- | --- | --- | --- |
| **Total EAT** | | | | | | |
| shape | Elongation | 0.842 | 0.079 | 0.814 | 0.090 | 0.011785646 |
| shape | Flatness | 0.000 | 0.000 | 0.000 | 0.000 | 0.24037101 |
| shape | LeastAxisLength | 0.000 | 0.000 | 0.000 | 0.000 | 0.332069788 |
| shape | MajorAxisLength | 163.938 | 27.015 | 223.750 | 29.232 | 7.71226E-22 |
| shape | Maximum2DDiameterColumn | 129.102 | 17.438 | 173.923 | 18.764 | 3.67619E-27 |
| shape | Maximum2DDiameterRow | 113.356 | 17.202 | 141.730 | 15.998 | 4.90621E-15 |
| shape | Maximum2DDiameterSlice | 135.702 | 19.385 | 180.199 | 18.716 | 4.96862E-25 |
| shape | Maximum3DDiameter | 135.702 | 19.385 | 180.199 | 18.716 | 4.96862E-25 |
| shape | MeshVolume | 371.515 | 244.588 | 394.236 | 354.537 | 0.197519266 |
| shape | MinorAxisLength | 137.354 | 21.051 | 180.615 | 23.025 | 4.20031E-18 |
| shape | Sphericity | 0.105 | 0.023 | 0.104 | 0.038 | 0.245309657 |
| shape | SurfaceArea | 2426.952 | 1289.523 | 2540.881 | 1191.424 | 0.600978945 |
| shape | SurfaceVolumeRatio | 7.360 | 2.412 | 8.335 | 3.747 | 0.729533372 |
| shape | VoxelVolume | 413.561 | 268.142 | 492.698 | 394.819 | 0.027200144 |
| first order | 10Percentile | -136.958 | 9.976 | -132.350 | 17.075 | 0.033264879 |
| first order | 90Percentile | -40.833 | 2.899 | -38.131 | 2.672 | 1.13746E-07 |
| first order | Energy | 84305480.100 | 52396855.290 | 39658214.057 | 28712984.241 | 1.32776E-09 |
| first order | Entropy | 3.157 | 0.115 | 3.062 | 0.196 | 0.001049421 |
| first order | InterquartileRange | 54.111 | 5.515 | 53.423 | 11.282 | 0.543271337 |
| first order | Kurtosis | 2.484 | 0.410 | 2.891 | 0.927 | 0.002254982 |
| first order | Maximum | -30.000 | 0.000 | -30.000 | 0.000 | #DZIEL/0! |
| first order | MeanAbsoluteDeviation | 29.694 | 2.449 | 29.486 | 4.925 | 0.58889011 |
| first order | Mean | -86.038 | 6.992 | -80.597 | 8.496 | 7.22348E-05 |
| first order | Median | -82.321 | 8.651 | -74.783 | 9.747 | 8.23874E-06 |
| first order | Minimum | -179.981 | 0.125 | -179.654 | 0.629 | 0.000373104 |
| first order | Range | 149.981 | 0.125 | 149.654 | 0.629 | 0.000247217 |
| first order | RobustMeanAbsoluteDeviation | 22.333 | 2.195 | 22.137 | 4.480 | 0.603958464 |
| first order | RootMeanSquared | 93.204 | 7.134 | 88.203 | 9.318 | 0.000480103 |
| first order | Skewness | -0.447 | 0.229 | -0.657 | 0.323 | 6.14873E-05 |
| first order | TotalEnergy | 3749722.495 | 2708447.195 | 3719677.635 | 3045689.870 | 0.45943891 |
| first order | Uniformity | 0.124 | 0.016 | 0.138 | 0.026 | 0.000254991 |
| first order | Variance | 1286.506 | 179.228 | 1300.760 | 349.493 | 0.98507641 |
| glcm | Autocorrelation | 47.689 | 5.733 | 51.228 | 8.695 | 0.00772107 |
| glcm | ClusterProminence | 713.380 | 131.247 | 596.883 | 226.340 | 7.77318E-05 |
| glcm | ClusterShade | -24.200 | 10.661 | -24.919 | 13.064 | 0.831203527 |
| glcm | ClusterTendency | 17.091 | 2.201 | 14.683 | 3.992 | 1.52794E-05 |
| glcm | Contrast | 3.382 | 1.042 | 6.095 | 2.994 | 1.05135E-08 |
| glcm | Correlation | 0.671 | 0.085 | 0.437 | 0.132 | 5.86163E-21 |
| glcm | DifferenceAverage | 1.312 | 0.232 | 1.790 | 0.503 | 4.95971E-09 |
| glcm | DifferenceEntropy | 2.098 | 0.189 | 2.421 | 0.315 | 1.18582E-09 |
| glcm | DifferenceVariance | 1.582 | 0.436 | 2.599 | 1.097 | 6.27919E-09 |
| glcm | Id | 0.565 | 0.043 | 0.500 | 0.067 | 1.58532E-08 |
| glcm | Idm | 0.519 | 0.054 | 0.439 | 0.083 | 1.48657E-08 |
| glcm | Idmn | 0.975 | 0.007 | 0.958 | 0.019 | 7.59076E-09 |
| glcm | Idn | 0.902 | 0.015 | 0.873 | 0.030 | 5.01259E-09 |
| glcm | Imc1 | -0.163 | 0.050 | -0.080 | 0.043 | 1.09406E-17 |
| glcm | Imc2 | 0.775 | 0.083 | 0.573 | 0.142 | 1.04337E-15 |
| glcm | InverseVariance | 0.455 | 0.026 | 0.400 | 0.055 | 1.21876E-09 |
| glcm | JointAverage | 6.637 | 0.457 | 6.973 | 0.637 | 0.00076754 |
| glcm | JointEnergy | 0.025 | 0.006 | 0.026 | 0.012 | 0.725389083 |
| glcm | JointEntropy | 5.738 | 0.252 | 5.836 | 0.525 | 0.250413532 |
| glcm | MCC | 0.686 | 0.084 | 0.469 | 0.132 | 5.11721E-19 |
| glcm | MaximumProbability | 0.048 | 0.013 | 0.055 | 0.030 | 0.151415842 |
| glcm | SumAverage | 13.273 | 0.914 | 13.946 | 1.274 | 0.00076754 |
| glcm | SumEntropy | 3.995 | 0.108 | 3.835 | 0.231 | 1.66198E-06 |
| glcm | SumSquares | 5.118 | 0.658 | 5.195 | 1.595 | 0.935280338 |
| gldm | DependenceEntropy | 5.539 | 0.236 | 4.982 | 0.280 | 3.77344E-22 |
| gldm | DependenceNonUniformity | 1984.745 | 1095.459 | 1468.277 | 1101.934 | 0.00057836 |
| gldm | DependenceNonUniformityNormalized | 0.215 | 0.033 | 0.302 | 0.087 | 5.67596E-10 |
| gldm | DependenceVariance | 2.214 | 0.591 | 1.395 | 0.620 | 2.54667E-11 |
| gldm | GrayLevelNonUniformity | 1127.919 | 555.941 | 587.737 | 332.180 | 1.84533E-11 |
| gldm | GrayLevelVariance | 5.729 | 0.787 | 5.776 | 1.534 | 0.925791667 |
| gldm | HighGrayLevelEmphasis | 52.073 | 5.952 | 57.281 | 7.109 | 1.15861E-05 |
| gldm | LargeDependenceEmphasis | 10.250 | 2.736 | 6.332 | 2.728 | 1.12183E-12 |
| gldm | LargeDependenceHighGrayLevelEmphasis | 526.450 | 147.991 | 379.190 | 180.556 | 3.04269E-06 |
| gldm | LargeDependenceLowGrayLevelEmphasis | 0.488 | 0.181 | 0.248 | 0.089 | 4.03538E-16 |
| gldm | LowGrayLevelEmphasis | 0.059 | 0.017 | 0.059 | 0.024 | 0.539734665 |
| gldm | SmallDependenceEmphasis | 0.319 | 0.072 | 0.479 | 0.127 | 1.98024E-13 |
| gldm | SmallDependenceHighGrayLevelEmphasis | 16.606 | 4.475 | 26.253 | 5.956 | 4.91465E-18 |
| gldm | SmallDependenceLowGrayLevelEmphasis | 0.024 | 0.009 | 0.036 | 0.019 | 0.000128317 |
| glrlm | GrayLevelNonUniformity | 850.401 | 408.720 | 496.620 | 289.568 | 1.7456E-09 |
| glrlm | GrayLevelNonUniformityNormalized | 0.121 | 0.015 | 0.135 | 0.024 | 0.0002144 |
| glrlm | GrayLevelVariance | 5.951 | 0.771 | 5.957 | 1.444 | 0.808154646 |
| glrlm | HighGrayLevelRunEmphasis | 52.169 | 5.646 | 57.037 | 6.889 | 1.46499E-05 |
| glrlm | LongRunEmphasis | 2.218 | 0.428 | 1.647 | 0.358 | 5.28188E-13 |
| glrlm | LongRunHighGrayLevelEmphasis | 114.833 | 24.323 | 96.097 | 28.028 | 0.000153882 |
| glrlm | LongRunLowGrayLevelEmphasis | 0.118 | 0.034 | 0.084 | 0.027 | 9.36359E-10 |
| glrlm | LowGrayLevelRunEmphasis | 0.062 | 0.016 | 0.061 | 0.024 | 0.417260153 |
| glrlm | RunEntropy | 4.148 | 0.199 | 3.761 | 0.165 | 4.17518E-22 |
| glrlm | RunLengthNonUniformity | 4687.098 | 2441.154 | 3113.144 | 2124.114 | 5.29537E-06 |
| glrlm | RunLengthNonUniformityNormalized | 0.661 | 0.056 | 0.758 | 0.081 | 8.62096E-12 |
| glrlm | RunPercentage | 0.774 | 0.048 | 0.853 | 0.057 | 2.81896E-13 |
| glrlm | RunVariance | 0.516 | 0.205 | 0.249 | 0.151 | 1.88533E-13 |
| glrlm | ShortRunEmphasis | 0.838 | 0.033 | 0.891 | 0.043 | 1.02465E-11 |
| glrlm | ShortRunHighGrayLevelEmphasis | 43.831 | 5.168 | 50.519 | 5.260 | 2.15084E-11 |
| glrlm | ShortRunLowGrayLevelEmphasis | 0.054 | 0.015 | 0.057 | 0.024 | 0.775314916 |
| glszm | GrayLevelNonUniformity | 371.541 | 187.537 | 311.046 | 209.873 | 0.009971723 |
| glszm | GrayLevelNonUniformityNormalized | 0.113 | 0.013 | 0.125 | 0.017 | 9.81561E-06 |
| glszm | GrayLevelVariance | 6.861 | 0.731 | 6.572 | 1.118 | 0.069188897 |
| glszm | HighGrayLevelZoneEmphasis | 51.945 | 4.851 | 56.054 | 6.231 | 1.77844E-05 |
| glszm | LargeAreaEmphasis | 28.936 | 22.398 | 9.878 | 11.307 | 2.06148E-08 |
| glszm | LargeAreaHighGrayLevelEmphasis | 1443.433 | 1090.030 | 590.951 | 710.699 | 5.92449E-07 |
| glszm | LargeAreaLowGrayLevelEmphasis | 1.155 | 0.812 | 0.319 | 0.221 | 6.71863E-12 |
| glszm | LowGrayLevelZoneEmphasis | 0.076 | 0.017 | 0.069 | 0.023 | 0.024691729 |
| glszm | SizeZoneNonUniformity | 1121.580 | 635.807 | 1327.664 | 1130.098 | 0.670984935 |
| glszm | SizeZoneNonUniformityNormalized | 0.346 | 0.067 | 0.476 | 0.107 | 1.42039E-12 |
| glszm | SmallAreaEmphasis | 0.605 | 0.061 | 0.710 | 0.079 | 1.5921E-13 |
| glszm | SmallAreaHighGrayLevelEmphasis | 31.286 | 4.124 | 38.974 | 4.150 | 1.05889E-19 |
| glszm | SmallAreaLowGrayLevelEmphasis | 0.052 | 0.015 | 0.055 | 0.021 | 0.519604337 |
| glszm | ZoneEntropy | 5.527 | 0.376 | 4.787 | 0.370 | 7.38249E-21 |
| glszm | ZonePercentage | 0.375 | 0.090 | 0.555 | 0.132 | 2.31395E-14 |
| glszm | ZoneVariance | 20.441 | 18.212 | 5.929 | 9.070 | 1.10786E-07 |
| ngtdm | Busyness | 13.505 | 7.873 | 10.662 | 8.164 | 0.005761221 |
| ngtdm | Coarseness | 0.001 | 0.001 | 0.003 | 0.003 | 1.59223E-05 |
| ngtdm | Complexity | 42.170 | 10.230 | 67.361 | 17.395 | 3.28825E-16 |
| ngtdm | Contrast | 0.096 | 0.031 | 0.157 | 0.082 | 1.34641E-06 |
| ngtdm | Strength | 0.056 | 0.025 | 0.142 | 0.147 | 5.56281E-06 |
| **LV EAT** | | | | | | |
| shape | Elongation | 0.307 | 0.069 | 0.355 | 0.069 | 0.000146969 |
| shape | Flatness | 0.000 | 0.000 | 0.000 | 0.000 | 0.678721968 |
| shape | LeastAxisLength | 0.000 | 0.000 | 0.000 | 0.000 | 0.025113365 |
| shape | MajorAxisLength | 131.747 | 22.589 | 172.985 | 26.089 | 7.28569E-15 |
| shape | Maximum2DDiameterColumn | 24.801 | 10.064 | 42.757 | 20.610 | 1.91804E-08 |
| shape | Maximum2DDiameterRow | 80.501 | 18.505 | 95.429 | 20.134 | 0.000176739 |
| shape | Maximum2DDiameterSlice | 95.607 | 15.450 | 125.441 | 15.561 | 1.09085E-17 |
| shape | Maximum3DDiameter | 95.607 | 15.450 | 125.441 | 15.561 | 1.09085E-17 |
| shape | MeshVolume | 92.536 | 72.481 | 100.008 | 102.864 | 0.24960495 |
| shape | MinorAxisLength | 40.666 | 11.455 | 61.580 | 16.314 | 1.31059E-12 |
| shape | Sphericity | 0.161 | 0.034 | 0.156 | 0.050 | 0.51922575 |
| shape | SurfaceArea | 622.998 | 380.107 | 657.552 | 373.446 | 0.62870716 |
| shape | SurfaceVolumeRatio | 7.790 | 2.481 | 8.823 | 3.770 | 0.636098675 |
| shape | VoxelVolume | 104.281 | 77.663 | 128.820 | 115.329 | 0.034853008 |
| first order | 10Percentile | -143.676 | 10.151 | -138.505 | 16.397 | 0.031869972 |
| first order | 90Percentile | -42.726 | 3.556 | -38.830 | 2.828 | 4.58729E-10 |
| first order | Energy | 23490182.271 | 16642396.312 | 10208130.346 | 7921668.384 | 6.29348E-09 |
| first order | Entropy | 3.207 | 0.107 | 3.114 | 0.187 | 0.000788533 |
| first order | InterquartileRange | 56.557 | 6.098 | 56.619 | 11.080 | 0.884434421 |
| first order | Kurtosis | 2.361 | 0.384 | 2.653 | 0.769 | 0.007278877 |
| first order | Maximum | -30.000 | 0.000 | -30.019 | 0.147 | 0.109333045 |
| first order | MeanAbsoluteDeviation | 30.946 | 2.587 | 31.045 | 4.742 | 0.924942943 |
| first order | Mean | -90.400 | 7.342 | -83.675 | 8.661 | 3.99889E-06 |
| first order | Median | -87.131 | 9.143 | -77.931 | 10.013 | 1.34989E-07 |
| first order | Minimum | -179.838 | 0.592 | -178.270 | 4.458 | 0.017339629 |
| first order | Range | 149.838 | 0.592 | 148.252 | 4.466 | 0.014603256 |
| first order | RobustMeanAbsoluteDeviation | 23.373 | 2.413 | 23.501 | 4.375 | 0.940983006 |
| first order | RootMeanSquared | 97.771 | 7.407 | 91.667 | 9.434 | 6.35316E-05 |
| first order | Skewness | -0.366 | 0.233 | -0.586 | 0.297 | 3.5306E-06 |
| first order | TotalEnergy | 1021454.717 | 793259.670 | 1026802.261 | 905280.947 | 0.447995076 |
| first order | Uniformity | 0.118 | 0.015 | 0.132 | 0.025 | 0.000317007 |
| first order | Variance | 1387.980 | 192.179 | 1418.713 | 338.520 | 0.562834297 |
| glcm | Autocorrelation | 44.884 | 5.777 | 48.755 | 7.534 | 0.000234012 |
| glcm | ClusterProminence | 713.173 | 125.013 | 616.811 | 199.932 | 0.000203812 |
| glcm | ClusterShade | -19.196 | 11.021 | -24.397 | 13.046 | 0.01618688 |
| glcm | ClusterTendency | 17.251 | 2.159 | 15.046 | 3.543 | 5.54065E-06 |
| glcm | Contrast | 4.433 | 1.587 | 7.328 | 3.248 | 1.25051E-08 |
| glcm | Correlation | 0.595 | 0.120 | 0.368 | 0.145 | 1.87255E-17 |
| glcm | DifferenceAverage | 1.502 | 0.291 | 1.979 | 0.515 | 1.14796E-08 |
| glcm | DifferenceEntropy | 2.248 | 0.224 | 2.518 | 0.292 | 7.53858E-08 |
| glcm | DifferenceVariance | 2.058 | 0.713 | 3.072 | 1.171 | 2.5685E-08 |
| glcm | Id | 0.537 | 0.044 | 0.478 | 0.063 | 5.224E-08 |
| glcm | Idm | 0.486 | 0.055 | 0.413 | 0.078 | 5.92326E-08 |
| glcm | Idmn | 0.968 | 0.010 | 0.950 | 0.020 | 4.73543E-09 |
| glcm | Idn | 0.891 | 0.018 | 0.862 | 0.030 | 4.82446E-09 |
| glcm | Imc1 | -0.140 | 0.048 | -0.099 | 0.062 | 7.13442E-05 |
| glcm | Imc2 | 0.738 | 0.084 | 0.620 | 0.127 | 3.04536E-08 |
| glcm | InverseVariance | 0.436 | 0.033 | 0.384 | 0.055 | 8.32675E-09 |
| glcm | JointAverage | 6.436 | 0.478 | 6.810 | 0.564 | 1.02502E-05 |
| glcm | JointEnergy | 0.023 | 0.005 | 0.025 | 0.012 | 0.054861127 |
| glcm | JointEntropy | 5.871 | 0.234 | 5.824 | 0.540 | 0.330404265 |
| glcm | MCC | 0.624 | 0.105 | 0.462 | 0.123 | 6.12631E-13 |
| glcm | MaximumProbability | 0.048 | 0.012 | 0.055 | 0.026 | 0.01854885 |
| glcm | SumAverage | 12.872 | 0.956 | 13.621 | 1.129 | 1.02502E-05 |
| glcm | SumEntropy | 4.000 | 0.105 | 3.813 | 0.243 | 2.64121E-08 |
| glcm | SumSquares | 5.421 | 0.695 | 5.593 | 1.532 | 0.694867747 |
| gldm | DependenceEntropy | 5.431 | 0.275 | 4.836 | 0.328 | 2.19603E-20 |
| gldm | DependenceNonUniformity | 531.423 | 308.312 | 368.161 | 249.813 | 6.8542E-05 |
| gldm | DependenceNonUniformityNormalized | 0.238 | 0.045 | 0.341 | 0.104 | 5.43875E-10 |
| gldm | DependenceVariance | 2.036 | 0.557 | 1.197 | 0.561 | 2.50662E-13 |
| gldm | GrayLevelNonUniformity | 276.898 | 169.594 | 143.820 | 97.121 | 1.39606E-08 |
| gldm | GrayLevelVariance | 6.178 | 0.841 | 6.291 | 1.481 | 0.627844556 |
| gldm | HighGrayLevelEmphasis | 48.720 | 6.129 | 54.041 | 5.979 | 7.6338E-07 |
| gldm | LargeDependenceEmphasis | 8.830 | 2.609 | 5.418 | 2.324 | 2.72892E-12 |
| gldm | LargeDependenceHighGrayLevelEmphasis | 426.894 | 117.870 | 312.671 | 145.625 | 1.50591E-05 |
| gldm | LargeDependenceLowGrayLevelEmphasis | 0.502 | 0.241 | 0.248 | 0.085 | 9.05895E-13 |
| gldm | LowGrayLevelEmphasis | 0.075 | 0.021 | 0.071 | 0.026 | 0.347420322 |
| gldm | SmallDependenceEmphasis | 0.380 | 0.097 | 0.530 | 0.133 | 4.1408E-11 |
| gldm | SmallDependenceHighGrayLevelEmphasis | 18.168 | 5.172 | 27.079 | 5.721 | 1.31472E-15 |
| gldm | SmallDependenceLowGrayLevelEmphasis | 0.037 | 0.015 | 0.048 | 0.023 | 0.000707998 |
| glrlm | GrayLevelNonUniformity | 212.766 | 124.251 | 121.368 | 79.668 | 9.19793E-08 |
| glrlm | GrayLevelNonUniformityNormalized | 0.115 | 0.015 | 0.128 | 0.024 | 0.000340807 |
| glrlm | GrayLevelVariance | 6.421 | 0.824 | 6.461 | 1.408 | 0.804592167 |
| glrlm | HighGrayLevelRunEmphasis | 48.640 | 5.798 | 53.631 | 5.711 | 1.50194E-06 |
| glrlm | LongRunEmphasis | 2.007 | 0.386 | 1.530 | 0.292 | 1.36389E-12 |
| glrlm | LongRunHighGrayLevelEmphasis | 97.307 | 19.119 | 84.509 | 22.059 | 0.001418203 |
| glrlm | LongRunLowGrayLevelEmphasis | 0.134 | 0.043 | 0.096 | 0.028 | 5.23516E-09 |
| glrlm | LowGrayLevelRunEmphasis | 0.079 | 0.021 | 0.074 | 0.026 | 0.251058445 |
| glrlm | RunEntropy | 4.070 | 0.210 | 3.709 | 0.173 | 4.14133E-19 |
| glrlm | RunLengthNonUniformity | 1269.090 | 708.450 | 779.132 | 528.528 | 7.11189E-07 |
| glrlm | RunLengthNonUniformityNormalized | 0.703 | 0.065 | 0.789 | 0.078 | 6.02727E-10 |
| glrlm | RunPercentage | 0.803 | 0.051 | 0.874 | 0.053 | 3.69154E-12 |
| glrlm | RunVariance | 0.428 | 0.179 | 0.201 | 0.119 | 9.31842E-14 |
| glrlm | ShortRunEmphasis | 0.862 | 0.036 | 0.907 | 0.039 | 6.44951E-10 |
| glrlm | ShortRunHighGrayLevelEmphasis | 41.918 | 5.476 | 48.238 | 4.351 | 3.62091E-11 |
| glrlm | ShortRunLowGrayLevelEmphasis | 0.070 | 0.019 | 0.070 | 0.026 | 0.913321352 |
| glszm | GrayLevelNonUniformity | 100.347 | 51.287 | 75.621 | 47.066 | 0.000277658 |
| glszm | GrayLevelNonUniformityNormalized | 0.108 | 0.013 | 0.120 | 0.019 | 0.000190439 |
| glszm | GrayLevelVariance | 7.252 | 0.781 | 7.009 | 1.179 | 0.256048111 |
| glszm | HighGrayLevelZoneEmphasis | 47.930 | 4.980 | 52.252 | 5.062 | 6.38024E-06 |
| glszm | LargeAreaEmphasis | 18.973 | 13.828 | 6.608 | 5.640 | 1.51287E-09 |
| glszm | LargeAreaHighGrayLevelEmphasis | 880.926 | 604.822 | 379.533 | 341.465 | 7.97875E-08 |
| glszm | LargeAreaLowGrayLevelEmphasis | 0.953 | 0.752 | 0.271 | 0.122 | 1.6182E-10 |
| glszm | LowGrayLevelZoneEmphasis | 0.093 | 0.021 | 0.083 | 0.025 | 0.018916954 |
| glszm | SizeZoneNonUniformity | 352.232 | 145.443 | 336.278 | 226.065 | 0.172804053 |
| glszm | SizeZoneNonUniformityNormalized | 0.407 | 0.096 | 0.520 | 0.121 | 1.27851E-08 |
| glszm | SmallAreaEmphasis | 0.656 | 0.080 | 0.741 | 0.085 | 1.20593E-08 |
| glszm | SmallAreaHighGrayLevelEmphasis | 31.033 | 4.546 | 37.680 | 3.695 | 3.48519E-15 |
| glszm | SmallAreaLowGrayLevelEmphasis | 0.069 | 0.019 | 0.069 | 0.024 | 0.712162929 |
| glszm | ZoneEntropy | 5.214 | 0.439 | 4.583 | 0.390 | 6.92044E-15 |
| glszm | ZonePercentage | 0.440 | 0.108 | 0.607 | 0.132 | 2.78355E-12 |
| glszm | ZoneVariance | 12.662 | 10.502 | 3.366 | 3.973 | 1.81621E-09 |
| ngtdm | Busyness | 4.085 | 2.258 | 2.840 | 2.027 | 9.25211E-05 |
| ngtdm | Coarseness | 0.005 | 0.002 | 0.009 | 0.008 | 1.17749E-05 |
| ngtdm | Complexity | 50.656 | 15.303 | 75.000 | 20.711 | 4.3636E-12 |
| ngtdm | Contrast | 0.128 | 0.050 | 0.197 | 0.095 | 1.0126E-06 |
| ngtdm | Strength | 0.195 | 0.091 | 0.428 | 0.376 | 1.1593E-06 |
| **RV EAT** | | | | | | |
| shape | Elongation | 0.239 | 0.057 | 0.203 | 0.054 | 1.51112E-05 |
| shape | Flatness | 0.000 | 0.000 | 0.000 | 0.000 | 0.461341102 |
| shape | LeastAxisLength | 0.000 | 0.000 | 0.000 | 0.000 | 0.030168881 |
| shape | MajorAxisLength | 114.426 | 20.464 | 150.766 | 29.437 | 5.95559E-13 |
| shape | Maximum2DDiameterColumn | 77.159 | 18.112 | 97.662 | 22.434 | 9.68457E-08 |
| shape | Maximum2DDiameterRow | 25.542 | 7.180 | 27.373 | 9.926 | 0.176206581 |
| shape | Maximum2DDiameterSlice | 90.205 | 17.059 | 116.887 | 19.121 | 6.0998E-13 |
| shape | Maximum3DDiameter | 90.205 | 17.059 | 116.887 | 19.121 | 6.0998E-13 |
| shape | MeshVolume | 159.878 | 109.781 | 164.638 | 148.246 | 0.388156688 |
| shape | MinorAxisLength | 26.917 | 6.500 | 29.996 | 8.146 | 0.12543085 |
| shape | Sphericity | 0.149 | 0.034 | 0.154 | 0.058 | 0.063213299 |
| shape | SurfaceArea | 978.483 | 564.575 | 955.109 | 448.142 | 0.682413546 |
| shape | SurfaceVolumeRatio | 6.958 | 2.357 | 7.740 | 3.581 | 0.961805778 |
| shape | VoxelVolume | 171.913 | 117.595 | 191.468 | 159.486 | 0.151996612 |
| first order | 10Percentile | -133.711 | 11.977 | -126.936 | 21.862 | 0.021476048 |
| first order | 90Percentile | -42.885 | 4.522 | -40.052 | 4.689 | 0.000918712 |
| first order | Energy | 35435312.414 | 24351303.922 | 14910183.698 | 11144182.989 | 1.10615E-09 |
| first order | Entropy | 3.104 | 0.137 | 2.954 | 0.308 | 0.000616357 |
| first order | InterquartileRange | 50.855 | 6.155 | 49.095 | 12.758 | 0.275169481 |
| first order | Kurtosis | 2.481 | 0.379 | 2.805 | 0.836 | 0.030205841 |
| first order | Maximum | -30.000 | 0.000 | -30.000 | 0.000 | #DZIEL/0! |
| first order | MeanAbsoluteDeviation | 28.028 | 2.907 | 27.076 | 6.120 | 0.179185882 |
| first order | Mean | -86.652 | 8.622 | -80.439 | 12.197 | 0.001362322 |
| first order | Median | -84.117 | 10.298 | -76.311 | 13.715 | 0.000806735 |
| first order | Minimum | -178.995 | 2.178 | -171.182 | 16.032 | 0.000107791 |
| first order | Range | 148.995 | 2.178 | 141.182 | 16.027 | 0.000105467 |
| first order | RobustMeanAbsoluteDeviation | 21.005 | 2.444 | 20.307 | 5.115 | 0.271394844 |
| first order | RootMeanSquared | 93.062 | 8.798 | 86.955 | 13.315 | 0.002610899 |
| first order | Skewness | -0.355 | 0.251 | -0.517 | 0.377 | 0.025597139 |
| first order | TotalEnergy | 1595992.627 | 1267189.131 | 1432977.175 | 1334338.619 | 0.854077622 |
| first order | Uniformity | 0.128 | 0.017 | 0.151 | 0.042 | 0.000373852 |
| first order | Variance | 1155.310 | 213.454 | 1121.967 | 430.877 | 0.369754607 |
| glcm | Autocorrelation | 46.759 | 6.559 | 45.805 | 10.595 | 0.334034082 |
| glcm | ClusterProminence | 656.338 | 165.015 | 503.305 | 248.991 | 2.22023E-05 |
| glcm | ClusterShade | -19.506 | 12.437 | -18.263 | 12.724 | 0.237211516 |
| glcm | ClusterTendency | 16.300 | 2.675 | 13.309 | 4.594 | 1.22417E-05 |
| glcm | Contrast | 2.691 | 1.060 | 5.429 | 3.268 | 1.02085E-07 |
| glcm | Correlation | 0.719 | 0.090 | 0.453 | 0.155 | 6.68918E-20 |
| glcm | DifferenceAverage | 1.191 | 0.249 | 1.685 | 0.556 | 3.44762E-08 |
| glcm | DifferenceEntropy | 1.952 | 0.218 | 2.303 | 0.372 | 1.8128E-08 |
| glcm | DifferenceVariance | 1.180 | 0.419 | 2.210 | 1.194 | 6.65724E-08 |
| glcm | Id | 0.581 | 0.047 | 0.513 | 0.076 | 9.71242E-08 |
| glcm | Idm | 0.539 | 0.059 | 0.455 | 0.094 | 7.69306E-08 |
| glcm | Idmn | 0.980 | 0.007 | 0.960 | 0.020 | 7.38612E-10 |
| glcm | Idn | 0.909 | 0.016 | 0.876 | 0.031 | 8.46822E-11 |
| glcm | Imc1 | -0.195 | 0.056 | -0.099 | 0.056 | 2.92418E-16 |
| glcm | Imc2 | 0.809 | 0.079 | 0.608 | 0.138 | 4.44116E-16 |
| glcm | InverseVariance | 0.470 | 0.027 | 0.409 | 0.062 | 2.7992E-09 |
| glcm | JointAverage | 6.562 | 0.523 | 6.548 | 0.825 | 0.650172012 |
| glcm | JointEnergy | 0.028 | 0.007 | 0.032 | 0.021 | 0.271633628 |
| glcm | JointEntropy | 5.561 | 0.313 | 5.614 | 0.693 | 0.633640745 |
| glcm | MCC | 0.731 | 0.087 | 0.499 | 0.146 | 3.57389E-18 |
| glcm | MaximumProbability | 0.054 | 0.015 | 0.065 | 0.044 | 0.132643755 |
| glcm | SumAverage | 13.123 | 1.045 | 13.096 | 1.650 | 0.650172012 |
| glcm | SumEntropy | 3.961 | 0.131 | 3.732 | 0.326 | 1.89892E-06 |
| glcm | SumSquares | 4.748 | 0.797 | 4.685 | 1.779 | 0.581071635 |
| gldm | DependenceEntropy | 5.511 | 0.220 | 4.941 | 0.319 | 7.23357E-21 |
| gldm | DependenceNonUniformity | 800.019 | 467.196 | 487.966 | 330.900 | 7.50927E-07 |
| gldm | DependenceNonUniformityNormalized | 0.211 | 0.029 | 0.277 | 0.073 | 1.33058E-08 |
| gldm | DependenceVariance | 2.214 | 0.536 | 1.482 | 0.620 | 1.64589E-10 |
| gldm | GrayLevelNonUniformity | 479.111 | 248.023 | 223.900 | 122.437 | 9.03563E-13 |
| gldm | GrayLevelVariance | 5.158 | 0.938 | 4.995 | 1.893 | 0.340768776 |
| gldm | HighGrayLevelEmphasis | 50.734 | 6.921 | 51.404 | 10.672 | 0.922522868 |
| gldm | LargeDependenceEmphasis | 11.421 | 3.091 | 7.346 | 3.316 | 1.40594E-10 |
| gldm | LargeDependenceHighGrayLevelEmphasis | 563.444 | 169.180 | 371.167 | 159.395 | 5.63183E-10 |
| gldm | LargeDependenceLowGrayLevelEmphasis | 0.540 | 0.221 | 0.344 | 0.207 | 1.41447E-06 |
| gldm | LowGrayLevelEmphasis | 0.054 | 0.020 | 0.060 | 0.028 | 0.280753566 |
| gldm | SmallDependenceEmphasis | 0.267 | 0.073 | 0.418 | 0.129 | 1.04133E-11 |
| gldm | SmallDependenceHighGrayLevelEmphasis | 14.006 | 4.118 | 21.320 | 6.465 | 7.86742E-11 |
| gldm | SmallDependenceLowGrayLevelEmphasis | 0.017 | 0.009 | 0.031 | 0.021 | 4.87874E-05 |
| glrlm | GrayLevelNonUniformity | 349.695 | 177.318 | 182.567 | 98.842 | 2.50406E-11 |
| glrlm | GrayLevelNonUniformityNormalized | 0.126 | 0.016 | 0.147 | 0.039 | 0.000324911 |
| glrlm | GrayLevelVariance | 5.306 | 0.942 | 5.125 | 1.867 | 0.288552493 |
| glrlm | HighGrayLevelRunEmphasis | 51.131 | 6.606 | 51.387 | 10.323 | 0.917142789 |
| glrlm | LongRunEmphasis | 2.421 | 0.514 | 1.799 | 0.483 | 1.97444E-10 |
| glrlm | LongRunHighGrayLevelEmphasis | 121.339 | 29.736 | 91.330 | 26.723 | 9.00011E-09 |
| glrlm | LongRunLowGrayLevelEmphasis | 0.120 | 0.043 | 0.097 | 0.042 | 0.001983253 |
| glrlm | LowGrayLevelRunEmphasis | 0.055 | 0.020 | 0.061 | 0.028 | 0.299976923 |
| glrlm | RunEntropy | 4.172 | 0.201 | 3.725 | 0.230 | 1.36897E-21 |
| glrlm | RunLengthNonUniformity | 1778.743 | 1035.878 | 1061.540 | 694.605 | 2.91066E-07 |
| glrlm | RunLengthNonUniformityNormalized | 0.625 | 0.061 | 0.723 | 0.091 | 7.66604E-10 |
| glrlm | RunPercentage | 0.750 | 0.054 | 0.830 | 0.066 | 3.64244E-11 |
| glrlm | RunVariance | 0.594 | 0.239 | 0.305 | 0.200 | 2.13021E-11 |
| glrlm | ShortRunEmphasis | 0.817 | 0.038 | 0.871 | 0.051 | 2.48895E-09 |
| glrlm | ShortRunHighGrayLevelEmphasis | 42.062 | 5.617 | 44.863 | 8.980 | 0.07832946 |
| glrlm | ShortRunLowGrayLevelEmphasis | 0.046 | 0.018 | 0.055 | 0.027 | 0.068159883 |
| glszm | GrayLevelNonUniformity | 134.774 | 77.331 | 103.348 | 60.573 | 0.000661679 |
| glszm | GrayLevelNonUniformityNormalized | 0.119 | 0.014 | 0.136 | 0.032 | 8.06329E-05 |
| glszm | GrayLevelVariance | 6.004 | 0.942 | 5.559 | 1.746 | 0.026446309 |
| glszm | HighGrayLevelZoneEmphasis | 52.223 | 5.967 | 51.189 | 9.271 | 0.42066641 |
| glszm | LargeAreaEmphasis | 42.219 | 37.354 | 15.355 | 24.786 | 3.59446E-06 |
| glszm | LargeAreaHighGrayLevelEmphasis | 2007.688 | 1767.543 | 678.659 | 827.338 | 2.06672E-07 |
| glszm | LargeAreaLowGrayLevelEmphasis | 1.653 | 1.399 | 0.672 | 1.229 | 4.44864E-05 |
| glszm | LowGrayLevelZoneEmphasis | 0.062 | 0.022 | 0.066 | 0.028 | 0.674280175 |
| glszm | SizeZoneNonUniformity | 327.775 | 261.017 | 386.255 | 316.451 | 0.804467499 |
| glszm | SizeZoneNonUniformityNormalized | 0.276 | 0.067 | 0.411 | 0.112 | 8.12206E-12 |
| glszm | SmallAreaEmphasis | 0.532 | 0.070 | 0.655 | 0.094 | 1.79259E-12 |
| glszm | SmallAreaHighGrayLevelEmphasis | 28.457 | 4.168 | 33.506 | 6.277 | 1.51621E-06 |
| glszm | SmallAreaLowGrayLevelEmphasis | 0.035 | 0.017 | 0.048 | 0.025 | 0.011524004 |
| glszm | ZoneEntropy | 5.695 | 0.393 | 4.829 | 0.381 | 2.29889E-24 |
| glszm | ZonePercentage | 0.320 | 0.093 | 0.497 | 0.140 | 1.06937E-12 |
| glszm | ZoneVariance | 29.406 | 29.305 | 9.696 | 19.663 | 1.27522E-05 |
| ngtdm | Busyness | 4.983 | 3.570 | 4.055 | 2.872 | 0.016564891 |
| ngtdm | Coarseness | 0.004 | 0.002 | 0.008 | 0.008 | 0.000113817 |
| ngtdm | Complexity | 34.738 | 9.610 | 57.434 | 22.115 | 4.33674E-10 |
| ngtdm | Contrast | 0.071 | 0.029 | 0.133 | 0.086 | 3.0463E-06 |
| ngtdm | Strength | 0.174 | 0.107 | 0.373 | 0.485 | 0.001513071 |
| **Atrial EAT** | | | | | | |
| shape | Elongation | 0.319 | 0.074 | 0.347 | 0.077 | 0.175701045 |
| shape | Flatness | 0.000 | 0.000 | 0.000 | 0.000 | 0.680084967 |
| shape | LeastAxisLength | 0.000 | 0.000 | 0.000 | 0.000 | 0.048612594 |
| shape | MajorAxisLength | 97.432 | 21.829 | 140.760 | 31.569 | 1.06895E-13 |
| shape | Maximum2DDiameterColumn | 28.479 | 13.810 | 41.772 | 23.356 | 0.005543983 |
| shape | Maximum2DDiameterRow | 55.764 | 18.600 | 88.859 | 31.100 | 8.74178E-11 |
| shape | Maximum2DDiameterSlice | 99.692 | 14.984 | 128.488 | 19.175 | 1.1593E-14 |
| shape | Maximum3DDiameter | 99.692 | 14.984 | 128.488 | 19.175 | 1.1593E-14 |
| shape | MeshVolume | 51.717 | 41.806 | 65.754 | 69.952 | 0.062494555 |
| shape | MinorAxisLength | 31.022 | 9.589 | 49.489 | 17.369 | 1.39902E-09 |
| shape | Sphericity | 0.176 | 0.033 | 0.168 | 0.057 | 0.766818333 |
| shape | SurfaceArea | 384.252 | 240.113 | 475.132 | 270.170 | 0.06782887 |
| shape | SurfaceVolumeRatio | 8.562 | 2.522 | 9.251 | 3.825 | 0.994576975 |
| shape | VoxelVolume | 61.842 | 45.841 | 89.539 | 77.928 | 0.002671857 |
| first order | 10Percentile | -139.029 | 12.168 | -139.602 | 13.979 | 0.675107643 |
| first order | 90Percentile | -38.425 | 2.668 | -37.848 | 3.085 | 0.327562385 |
| first order | Energy | 11851337.605 | 8161909.623 | 7779803.358 | 6298300.883 | 0.000191834 |
| first order | Entropy | 3.132 | 0.144 | 3.098 | 0.181 | 0.23811633 |
| first order | InterquartileRange | 56.114 | 8.518 | 57.787 | 11.289 | 0.349048361 |
| first order | Kurtosis | 2.653 | 0.658 | 2.624 | 0.724 | 0.860159163 |
| first order | Maximum | -30.000 | 0.000 | -30.038 | 0.121 | 0.010380177 |
| first order | MeanAbsoluteDeviation | 31.163 | 3.418 | 31.843 | 4.373 | 0.279895855 |
| first order | Mean | -82.869 | 7.685 | -82.165 | 8.303 | 0.635673467 |
| first order | Median | -76.293 | 9.653 | -74.704 | 10.941 | 0.359799779 |
| first order | Minimum | -179.724 | 0.664 | -178.182 | 4.079 | 0.007181077 |
| first order | Range | 149.724 | 0.664 | 148.145 | 4.098 | 0.00567736 |
| first order | RobustMeanAbsoluteDeviation | 23.346 | 3.298 | 24.026 | 4.299 | 0.307311852 |
| first order | RootMeanSquared | 91.037 | 8.036 | 90.677 | 8.610 | 0.854271101 |
| first order | Skewness | -0.636 | 0.277 | -0.644 | 0.323 | 0.792063184 |
| first order | TotalEnergy | 526062.927 | 447697.543 | 717599.418 | 605563.323 | 0.008858427 |
| first order | Uniformity | 0.131 | 0.023 | 0.137 | 0.030 | 0.182538963 |
| first order | Variance | 1425.791 | 246.684 | 1478.742 | 318.392 | 0.216984922 |
| glcm | Autocorrelation | 51.940 | 6.735 | 51.606 | 10.168 | 0.998039142 |
| glcm | ClusterProminence | 727.234 | 164.918 | 643.980 | 219.344 | 0.004656489 |
| glcm | ClusterShade | -33.502 | 12.152 | -28.112 | 16.099 | 0.031162675 |
| glcm | ClusterTendency | 16.556 | 2.653 | 15.273 | 3.794 | 0.008814433 |
| glcm | Contrast | 5.141 | 1.884 | 7.477 | 3.308 | 6.81392E-07 |
| glcm | Correlation | 0.531 | 0.115 | 0.352 | 0.129 | 1.69036E-15 |
| glcm | DifferenceAverage | 1.593 | 0.307 | 1.989 | 0.507 | 1.71663E-07 |
| glcm | DifferenceEntropy | 2.324 | 0.212 | 2.518 | 0.288 | 2.18312E-05 |
| glcm | DifferenceVariance | 2.462 | 0.836 | 3.197 | 1.169 | 1.40381E-05 |
| glcm | Id | 0.532 | 0.042 | 0.481 | 0.065 | 4.78082E-07 |
| glcm | Idm | 0.478 | 0.052 | 0.416 | 0.079 | 4.38224E-07 |
| glcm | Idmn | 0.964 | 0.012 | 0.949 | 0.021 | 2.49339E-07 |
| glcm | Idn | 0.886 | 0.018 | 0.862 | 0.030 | 7.79882E-08 |
| glcm | Imc1 | -0.128 | 0.036 | -0.111 | 0.089 | 0.450988371 |
| glcm | Imc2 | 0.717 | 0.065 | 0.631 | 0.140 | 0.000228358 |
| glcm | InverseVariance | 0.422 | 0.037 | 0.377 | 0.053 | 7.82761E-08 |
| glcm | JointAverage | 6.983 | 0.510 | 7.000 | 0.714 | 0.646538623 |
| glcm | JointEnergy | 0.027 | 0.008 | 0.032 | 0.026 | 0.101749838 |
| glcm | JointEntropy | 5.759 | 0.275 | 5.697 | 0.717 | 0.243256744 |
| glcm | MCC | 0.584 | 0.085 | 0.470 | 0.133 | 1.23404E-07 |
| glcm | MaximumProbability | 0.063 | 0.024 | 0.070 | 0.054 | 0.185943543 |
| glcm | SumAverage | 13.966 | 1.021 | 14.000 | 1.427 | 0.646538623 |
| glcm | SumEntropy | 3.899 | 0.142 | 3.741 | 0.347 | 0.000183616 |
| glcm | SumSquares | 5.424 | 0.971 | 5.688 | 1.592 | 0.300725416 |
| gldm | DependenceEntropy | 5.216 | 0.288 | 4.726 | 0.349 | 4.49331E-16 |
| gldm | DependenceNonUniformity | 336.111 | 182.289 | 305.169 | 230.952 | 0.102119536 |
| gldm | DependenceNonUniformityNormalized | 0.258 | 0.058 | 0.355 | 0.102 | 1.72545E-10 |
| gldm | DependenceVariance | 1.929 | 0.565 | 1.147 | 0.582 | 1.19921E-12 |
| gldm | GrayLevelNonUniformity | 180.531 | 104.287 | 108.116 | 68.989 | 3.91138E-07 |
| gldm | GrayLevelVariance | 6.324 | 1.076 | 6.550 | 1.405 | 0.214104491 |
| gldm | HighGrayLevelEmphasis | 55.648 | 6.537 | 56.118 | 7.176 | 0.649476642 |
| gldm | LargeDependenceEmphasis | 7.927 | 2.374 | 4.999 | 2.023 | 1.89882E-12 |
| gldm | LargeDependenceHighGrayLevelEmphasis | 476.267 | 156.089 | 307.411 | 145.475 | 1.16015E-09 |
| gldm | LargeDependenceLowGrayLevelEmphasis | 0.329 | 0.135 | 0.223 | 0.086 | 5.82229E-08 |
| gldm | LowGrayLevelEmphasis | 0.067 | 0.022 | 0.070 | 0.024 | 0.354522003 |
| gldm | SmallDependenceEmphasis | 0.427 | 0.102 | 0.559 | 0.120 | 3.33042E-11 |
| gldm | SmallDependenceHighGrayLevelEmphasis | 21.719 | 5.075 | 29.282 | 5.673 | 4.54002E-14 |
| gldm | SmallDependenceLowGrayLevelEmphasis | 0.041 | 0.018 | 0.050 | 0.022 | 0.003566923 |
| glrlm | GrayLevelNonUniformity | 140.467 | 77.702 | 93.698 | 60.847 | 1.07065E-05 |
| glrlm | GrayLevelNonUniformityNormalized | 0.125 | 0.021 | 0.132 | 0.026 | 0.116871095 |
| glrlm | GrayLevelVariance | 6.612 | 1.038 | 6.726 | 1.322 | 0.429030976 |
| glrlm | HighGrayLevelRunEmphasis | 54.747 | 6.274 | 55.456 | 6.697 | 0.50776446 |
| glrlm | LongRunEmphasis | 1.869 | 0.347 | 1.473 | 0.246 | 3.351E-12 |
| glrlm | LongRunHighGrayLevelEmphasis | 107.294 | 24.630 | 85.104 | 21.338 | 9.59247E-08 |
| glrlm | LongRunLowGrayLevelEmphasis | 0.106 | 0.030 | 0.092 | 0.026 | 0.004395996 |
| glrlm | LowGrayLevelRunEmphasis | 0.072 | 0.022 | 0.073 | 0.024 | 0.65432188 |
| glrlm | RunEntropy | 3.925 | 0.201 | 3.649 | 0.182 | 2.06311E-14 |
| glrlm | RunLengthNonUniformity | 800.893 | 427.949 | 628.002 | 458.216 | 0.002914527 |
| glrlm | RunLengthNonUniformityNormalized | 0.729 | 0.063 | 0.806 | 0.068 | 1.02881E-10 |
| glrlm | RunPercentage | 0.823 | 0.047 | 0.885 | 0.046 | 1.35292E-12 |
| glrlm | RunVariance | 0.367 | 0.161 | 0.180 | 0.103 | 6.23827E-13 |
| glrlm | ShortRunEmphasis | 0.876 | 0.034 | 0.915 | 0.034 | 1.58398E-10 |
| glrlm | ShortRunHighGrayLevelEmphasis | 47.358 | 5.392 | 50.232 | 5.490 | 0.002082255 |
| glrlm | ShortRunLowGrayLevelEmphasis | 0.067 | 0.022 | 0.070 | 0.024 | 0.349475017 |
| glszm | GrayLevelNonUniformity | 69.764 | 35.324 | 62.803 | 43.564 | 0.067045267 |
| glszm | GrayLevelNonUniformityNormalized | 0.112 | 0.017 | 0.121 | 0.017 | 0.004847101 |
| glszm | GrayLevelVariance | 7.480 | 0.943 | 7.216 | 1.108 | 0.299312685 |
| glszm | HighGrayLevelZoneEmphasis | 51.707 | 5.653 | 53.439 | 5.611 | 0.093320046 |
| glszm | LargeAreaEmphasis | 15.270 | 14.507 | 5.455 | 5.656 | 2.44076E-06 |
| glszm | LargeAreaHighGrayLevelEmphasis | 932.252 | 858.280 | 340.418 | 328.191 | 1.12192E-06 |
| glszm | LargeAreaLowGrayLevelEmphasis | 0.534 | 0.407 | 0.232 | 0.137 | 1.37588E-07 |
| glszm | LowGrayLevelZoneEmphasis | 0.090 | 0.022 | 0.082 | 0.023 | 0.149841513 |
| glszm | SizeZoneNonUniformity | 266.622 | 107.278 | 299.516 | 230.097 | 0.809807839 |
| glszm | SizeZoneNonUniformityNormalized | 0.459 | 0.106 | 0.554 | 0.112 | 1.47278E-07 |
| glszm | SmallAreaEmphasis | 0.697 | 0.080 | 0.766 | 0.074 | 1.01791E-07 |
| glszm | SmallAreaHighGrayLevelEmphasis | 34.470 | 4.331 | 39.616 | 4.223 | 3.80324E-11 |
| glszm | SmallAreaLowGrayLevelEmphasis | 0.073 | 0.023 | 0.070 | 0.023 | 0.963825245 |
| glszm | ZoneEntropy | 4.944 | 0.410 | 4.437 | 0.367 | 6.21781E-13 |
| glszm | ZonePercentage | 0.489 | 0.109 | 0.635 | 0.116 | 1.83533E-12 |
| glszm | ZoneVariance | 10.220 | 11.641 | 2.722 | 4.508 | 7.62378E-06 |
| ngtdm | Busyness | 2.445 | 1.328 | 2.322 | 1.902 | 0.240410242 |
| ngtdm | Coarseness | 0.006 | 0.002 | 0.013 | 0.013 | 1.24324E-05 |
| ngtdm | Complexity | 60.162 | 16.524 | 76.988 | 18.025 | 1.75308E-08 |
| ngtdm | Contrast | 0.152 | 0.067 | 0.207 | 0.088 | 2.60197E-05 |
| ngtdm | Strength | 0.292 | 0.112 | 0.684 | 0.874 | 0.000159462 |

HFrEF, heart failure wih reduced ejection fraction; non-HF, non-heart failure; SD, standard deviation; glcm, Gray Level Co-occurrence Matrix; gldm, Gray Level Dependence Matrix; glrlm, Gray Level Run Length Matrix; glszm**,** Gray Level Size Zone Matrix; Neighbouring Gray Tone Difference Matrix. After Bonferroni correction for multiple comparisons, the significance level for t-test for radiomic parameters was 0.000481.

**Figure S1**

**
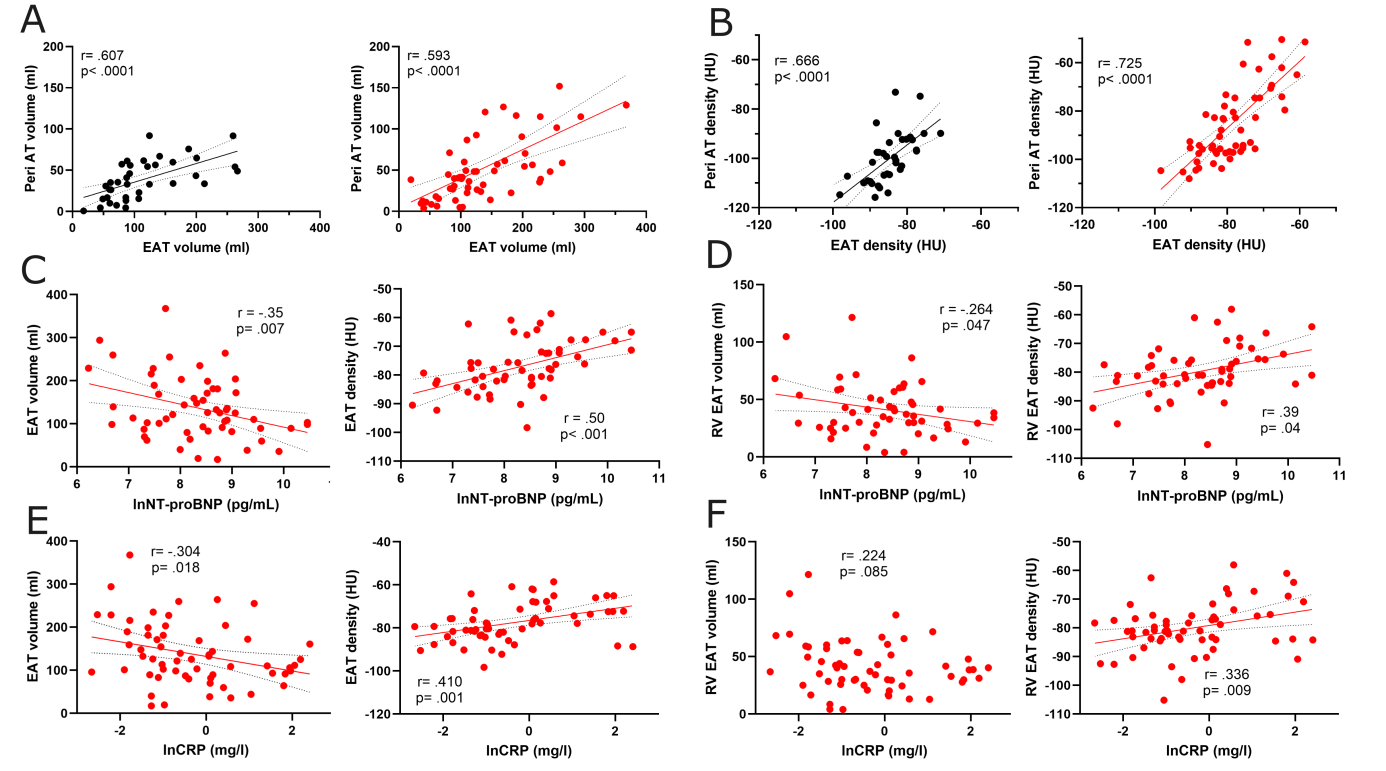
**

**Fig. S1 Correlation between epicardial adipose tissue characteristics and systemic metabolic, inflammatory, neurohormonal and other parameters in subjects with and without heart failure with reduced ejection fraction**

Correlation between pericardial adipose tissue (Peri AT) volume **[A]** and density **[B]** and total epicardial adipose tissue (EAT) volume or density, respectively. Correlation between total **[C]**, **[E]** and right ventricular (RV) **[D]**, **[F]** EAT volume and density, respectively and ln-transformed serum NTpro-BNP and C-reactive protein (CRP) concentrations in non-heart failure (non-HF, black dots and lines) subjects and heart failure with reduced ejection fraction (HFrEF, red dots and lines) patients. All panels display individual data points and the results of Pearson’s correlation analysis, with r indicating correlation strength and p indicating statistical significance for each association; dotted lines represent the confidence intervals.
